# Concentration-Dependent Effects of Foliar Zinc Oxide Nanoparticles on Growth, Photosynthesis, Antioxidant Defence and Yield in *Brassica juncea* L.

**DOI:** 10.3390/plants15203177

**Published:** 2026-10-16

**Authors:** Rizwana Kouser, Mohammad Shiraz, Sameen Zaidi, Shamsul Hayat, Andrzej Bajguz

**Affiliations:** 1 Department of Botany, Plant Physiology Section, Faculty of Life Sciences, Aligarh Muslim University, Aligarh 202002, India; rizwanakhan4461@gmail.com (R.K.); shirazamu@gmail.com (M.S.); sameenzaidi1179@gmail.com (S.Z.); 2 Department of Biology and Plant Ecology, Faculty of Biology, University of Bialystok, Konstantego Ciolkowskiego 1J, 15-245 Bialystok, Poland

**Keywords:** zinc oxide nanoparticles, photosynthesis, antioxidant, reactive oxygen species

## Abstract

Nanotechnology offers approaches to improve crop productivity through nutrient delivery. This study investigated the concentration-dependent effects of foliar-applied zinc oxide nanoparticles (ZnO-NPs) on growth, photosynthesis, antioxidant defense, redox homeostasis, and yield of mustard (*Brassica juncea* L.) ZnO-NPs were characterized by X-ray diffraction, Fourier transform infrared spectroscopy, and scanning electron microscopy, confirming a crystalline hexagonal wurtzite structure with a crystallite size of 29.3 nm and an aggregated morphology. Plants were treated with 25, 50, 75, or 100 mg/L ZnO-NPs and evaluated at 30 and 45 days after sowing, with yield assessed at harvest. Responses were concentration-dependent, with 50 mg/L producing the greatest benefits. This treatment enhanced growth, biomass, SPAD index, leaf area, gas exchange, PSII efficiency, carbohydrate and protein accumulation, antioxidant enzyme activities, leaf elemental composition, and yield traits. It also reduced reactive oxygen species, malondialdehyde, and electrolyte leakage while increasing proline and glutathione, indicating improved redox balance and membrane stability. Among the concentrations tested, 50 mg/L ZnO-NPs produced the most consistent beneficial response under the present experimental conditions, whereas the magnitude of these effects declined at 75 and 100 mg/L. These findings highlight the significance of concentration optimization while considering the use of ZnO-NPs as foliar treatment in *B. juncea* for Zn fertilization. Nevertheless, further comparative studies using conventional Zn fertilizers are necessary to determine whether the observed responses are specifically associated with the nanoscale properties of ZnO-NPs or are primarily attributable to Zn nutrition.

## 1. Introduction

Modern agriculture has found a transformative tool in nanotechnology, which provides new solutions to meet the increasing demand for food production while protecting natural resources. The agricultural industry has begun to adopt nanoparticles (NPs) as nanofertilizers and nanopesticides, enabling farmers to boost crop yields while optimizing resource use and operations in home agricultural systems and environmental settings [1,2]. Due to their extremely large surface area relative to volume, NPs have physicochemical characteristics that can be different from those of bulk materials and may influence interactions between NPs and plants [3]. Zinc oxide nanoparticles (ZnO-NPs) are such nanoparticles that have been studied for possible use in Zn nutrition [4]. But there is a need for control to differentiate these two aspects. They are also employed in nano-encapsulated pesticides and herbicides to enhance bioavailability and prolong residual activity while requiring lower application doses [5]. In addition, antimicrobial and photocatalytic NPs such as silver (Ag), titanium dioxide (TiO_2_), and ZnO-NPs are utilized for seed coating, pest management, and post-harvest protection [6,7,8]. Moreover, nano-sensors and nano-carriers enable real-time monitoring of soil and plant health and facilitate the delivery of growth regulators, genes, and other bioactive compounds to plants [9,10]. These advancements can significantly improve resource-use efficiency in agriculture and support the development of more sustainable and eco-friendly farming systems.

ZnO-NPs are among the most widely produced and utilized nanomaterials owing to their diverse industrial and agricultural applications. They typically occur as white, insoluble powders with particle sizes ranging from 1 to 100 nm. ZnO-NPs possess a wide band gap of 3.37 eV at room temperature, which contributes to their remarkable thermal, chemical, and electrical stability. ZnO-NPs are synthesized by several methods, including chemical and co-precipitation [11], sol–gel [12], hydrothermal [13], and thermal decomposition methods [14] and green synthesis using plant extracts or biopolymers, which is gaining interest for biomedical and environmental applications [15]. Although ZnO-NPs are increasingly being explored as an alternative means of supplying Zn to crop plants, their effects are highly dependent on the plant species, exposure level, nanoparticle characteristics and mode of application. Investigations conducted in *Brassica* and other crop species have reported enhanced growth, photosynthetic activity, nutrient accumulation and yield following ZnO-NP treatment; however, neutral or adverse responses have also been documented, particularly at elevated exposure levels. These contrasting outcomes indicate that plant responses to ZnO-NPs cannot be generalized and may arise from differences in particle properties, applied dose, delivery pathway, plant–nanoparticle interactions and species-specific physiological characteristics. High concentrations of ZnO-NPs may lead to phytotoxicity [16,17]. Previous studies have shown that ZnO-NPs applied at lower concentrations, such as 1–10 ppm in hydroponic systems or below 50 mg/L in soil, can positively influence root and shoot development, as well as enhance biomass, in various crops. On the contrary, higher concentrations (50 mg/L) have been reported to be linked to growth inhibition and low biomass production, implying that too much exposure of ZnO-NP can cause physiological stress and/or Zn toxicity [18,19,20,21]. Nevertheless, it is essential to make a distinction between the phytotoxicity of NPs and the toxic effects of high Zn concentration. ZnO-NPs improved plant height, leaf area, biomass, chlorophyll content, and photosynthesis efficiency in rice (*Oryza sativa*) and wheat (*Triticum aestivum*) plants [22,23,24]. Foliar application of ZnO-NPs has been reported to enhance grain quality, grain yield, and Zn enrichment in rice plants [25]. ZnO-NPs help plants withstand abiotic stress conditions by regulating key physiological and biochemical activities, leading to enhanced growth performance, higher productivity, and improved nutritional composition [22,26]. Additionally, ZnO-NPs are widely explored as nanofertilizers and nano-fortification materials because of their ability to improve nutrient utilization, increase crop productivity, and reduce zinc losses, especially under protected cultivation conditions [27,28]. However, excessive or prolonged application of ZnO-NPs may lead to the accumulation of zinc in soil and surrounding ecosystems, thereby increasing the risk of environmental toxicity and ecological imbalance [21].

Two major components of agriculture economy include cereals and oilseed crops. India ranks fourth in terms of production of oilseeds and is the largest importer of vegetable oils [29]. Rapeseed-mustard is an important oilseed crop which is grown widely in temperate and subtropical regions, including India and some parts of Europe [30]. *Brassica juncea* L., commonly known as Indian mustard, is a major oilseed crop from the family *Brassicaceae*. India is the third -largest producer of rapeseed and mustard next to Canada and China, having an approximate annual production of 13.26 million tonnes in 2023–2024. Rajasthan, Uttar Pradesh, Haryana, Madhya Pradesh, and Gujarat are major producers of *B. juncea* in India. *B. juncea* (Var. Varuna) is a high-yield Indian mustard variety that was developed in India, which matures early, has high oil content, and is adapted to the North Indian climate. However, the productivity of *B. juncea* is restrained by many biotic and abiotic stresses. In *B. juncea*, particularly when ZnO-NPs are applied to the foliage, the concentration-dependent responses of plants have not yet been fully characterized. Identifying an application level that can stimulate desirable physiological and growth responses without causing adverse effects is therefore important for optimizing ZnO-NP use in this crop [31].

To address this gap, the present study evaluated ZnO-NP treatments across a concentration range of 25–100 mg/L and assessed their effects on plant growth, photosynthetic performance, mineral profile, antioxidant defense and yield of *B. juncea*. A concentration series of 25, 50, 75 and 100 mg/L was selected to provide a gradient of treatment for a variety of responses that may be stimulating or inhibiting within the scope of the current study, and not for an ideal agricultural application rate. It was expected that moderate levels of ZnO-NPs would enhance plant functions, while high levels would inhibit them. To test this hypothesis, plants were exposed to these different concentrations of ZnO-NPs, and a comprehensive set of morphological, physiological, biochemical and yield-related traits was evaluated.

## 2. Results

### 2.1. Characterization of ZnO-NPs

**Figure 1:**
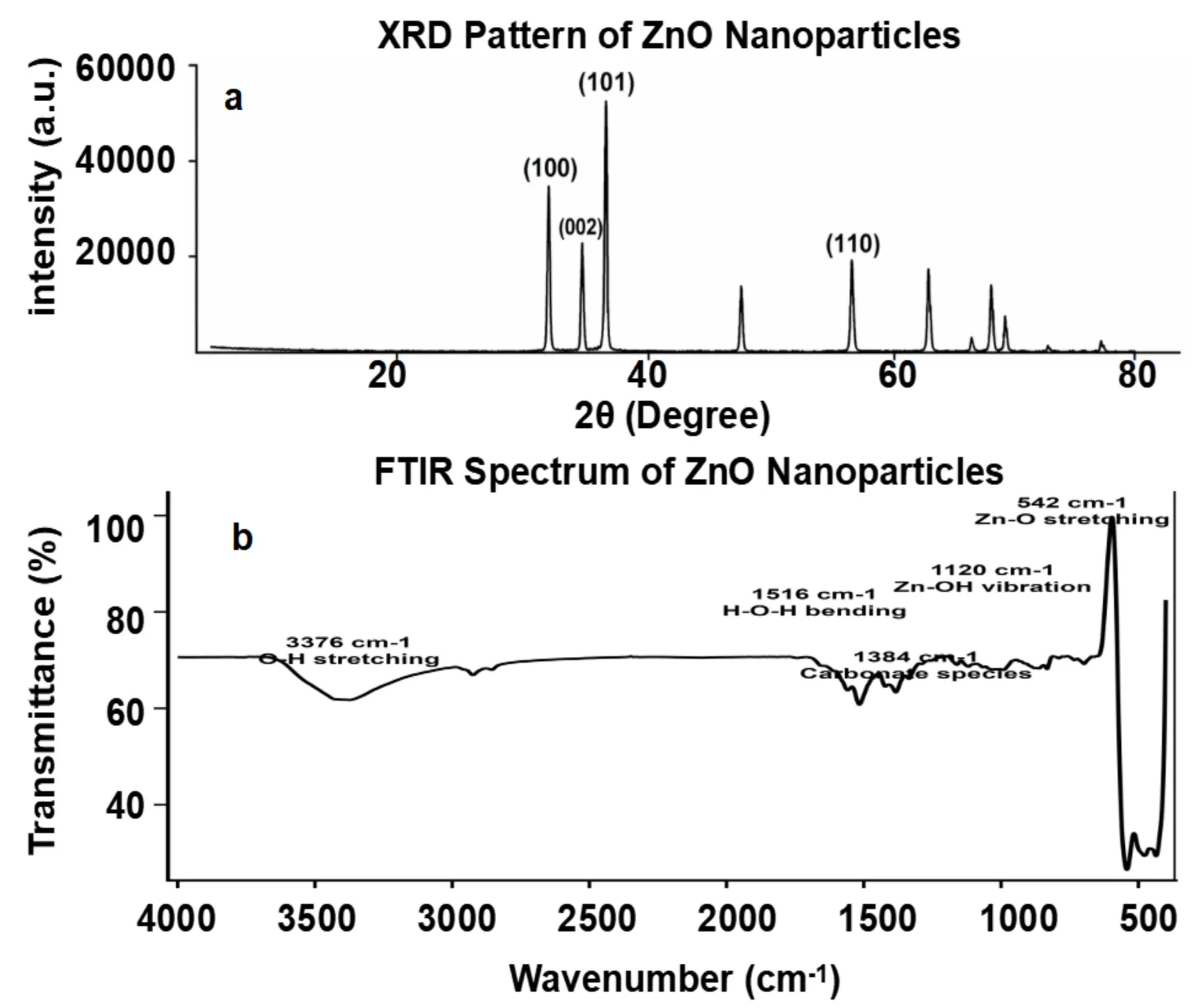
(**a**) X-ray diffraction (XRD) and (**b**) Fourier transform infrared spectroscopy (FTIR) of ZnO-NPs. The diffraction peaks correspond to the hexagonal wurtzite ZnO phase (JCPDS/PDF Card No. 36-1451). Characteristic FTIR bands were observed at 3376, 1516, 1384, 1120, and 542 cm^−1^, corresponding to O–H stretching, H–O–H bending, carbonate species, Zn–OH vibration, and Zn–O stretching, respectively.

XRD analysis was performed for the determination of the crystalline structure of the commercially available ZnO-NPs, as demonstrated in Figure 1a. The obtained results clearly revealed strong and sharp diffraction peaks at the 2θ angles of about 31.7°, 34.4°, 36.2°, 56.6°, and 62.8°, corresponding to the (100), (002), (101), (110), and (103) crystal plane indices, respectively, of the hexagonal wurtzite structure of ZnO-NPs [32]. The crystallite size was estimated to be 29.3 nm, and this value was confirmed to be similar to that obtained through Scherrer’s method.

FTIR spectroscopy was conducted to determine the type of functional groups and adsorbates available on the ZnO-NPs. The FTIR spectrum of the ZnO-NPs was recorded in the range of 450 to 4000 cm^−1^ shown in Figure 1b. The spectrum exhibited prominent absorption bands at 3376 cm^−1^ and 1516 cm^−1^, which were attributed to the stretching and bending vibrations of O-H and O-H-O groups, respectively, associated with water molecules [33]. The appearance of a characteristic band at 542 cm^−1^ confirmed the Zn-O stretching vibration, confirming the ZnO-NPs [32].

**Figure 2:**
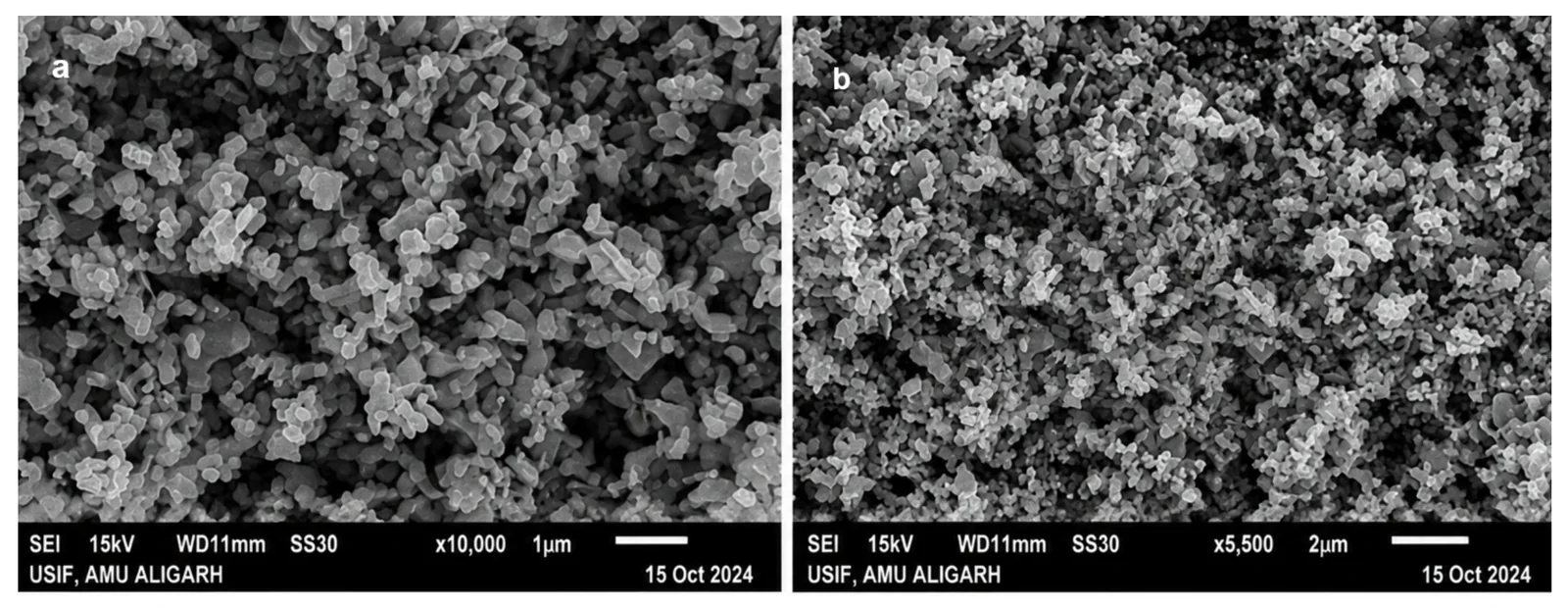
Scanning electron microscopy (SEM) images of commercially available ZnO-NPs that form agglomerates of irregular-shaped particles. The images suggest nanoparticle agglomeration and are meant to provide morphology information and not particle size determination. Scale bars: (**a**) 1 μm and (**b**) 2 μm.

Scanning electron microscopy (SEM) analysis of ZnO-NPs revealed irregularly shaped nanoparticles that aggregated to form clusters (Figure 2). At higher magnification (1 µm), non-uniform, quasi-spherical, elongated, and tightly packed nanoparticles were observed (Figure 2a).

### 2.2. Growth Characteristics

**Figure 3:**
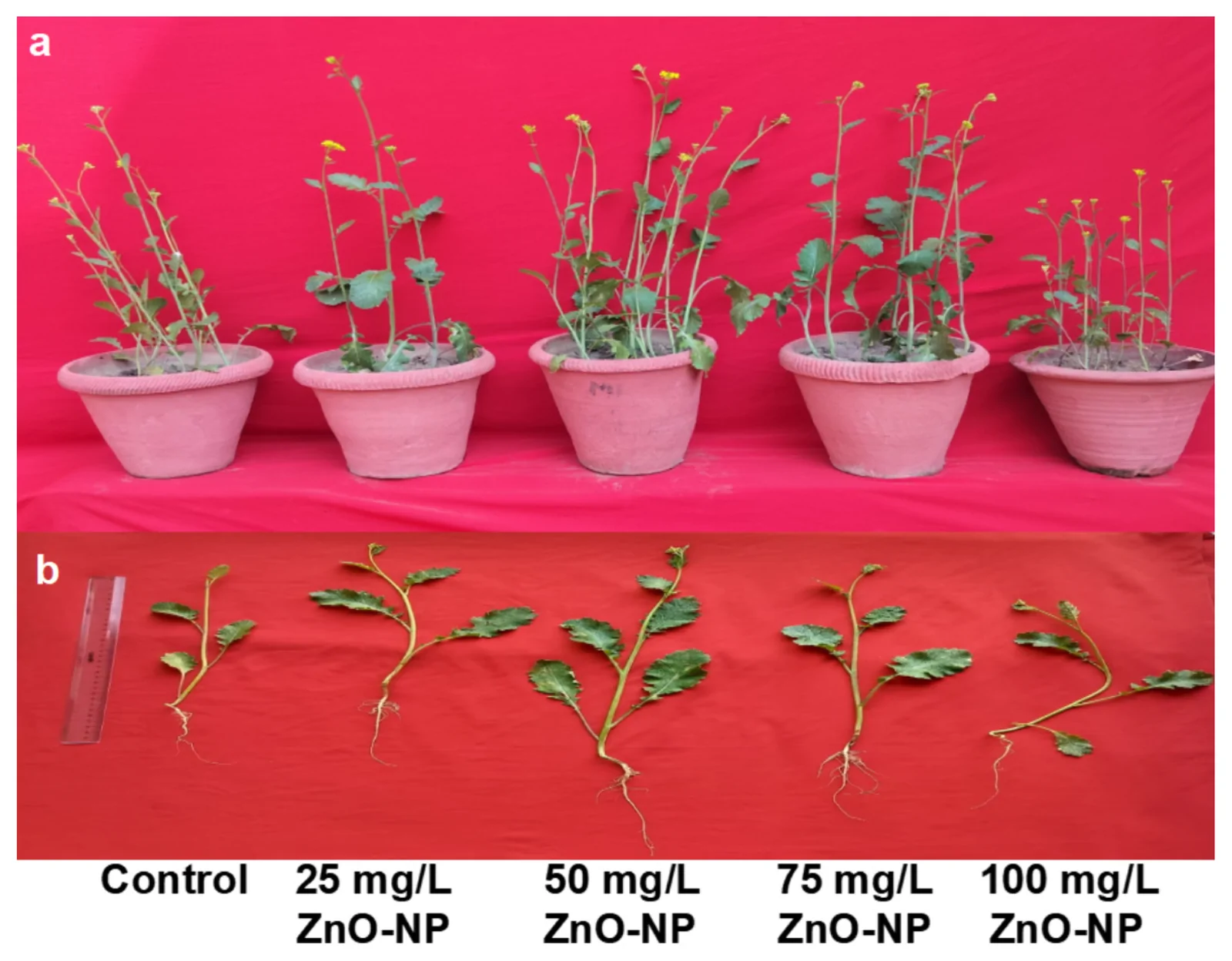
Representative *B. juncea* at 45 DAS with several ZnO-NP treatments (control, 25, 50, 75, and 100 mg/L). (**a**) Whole potted plants and (**b**) uprooted plant showing root and shoot morphology.

**Figure 4:**
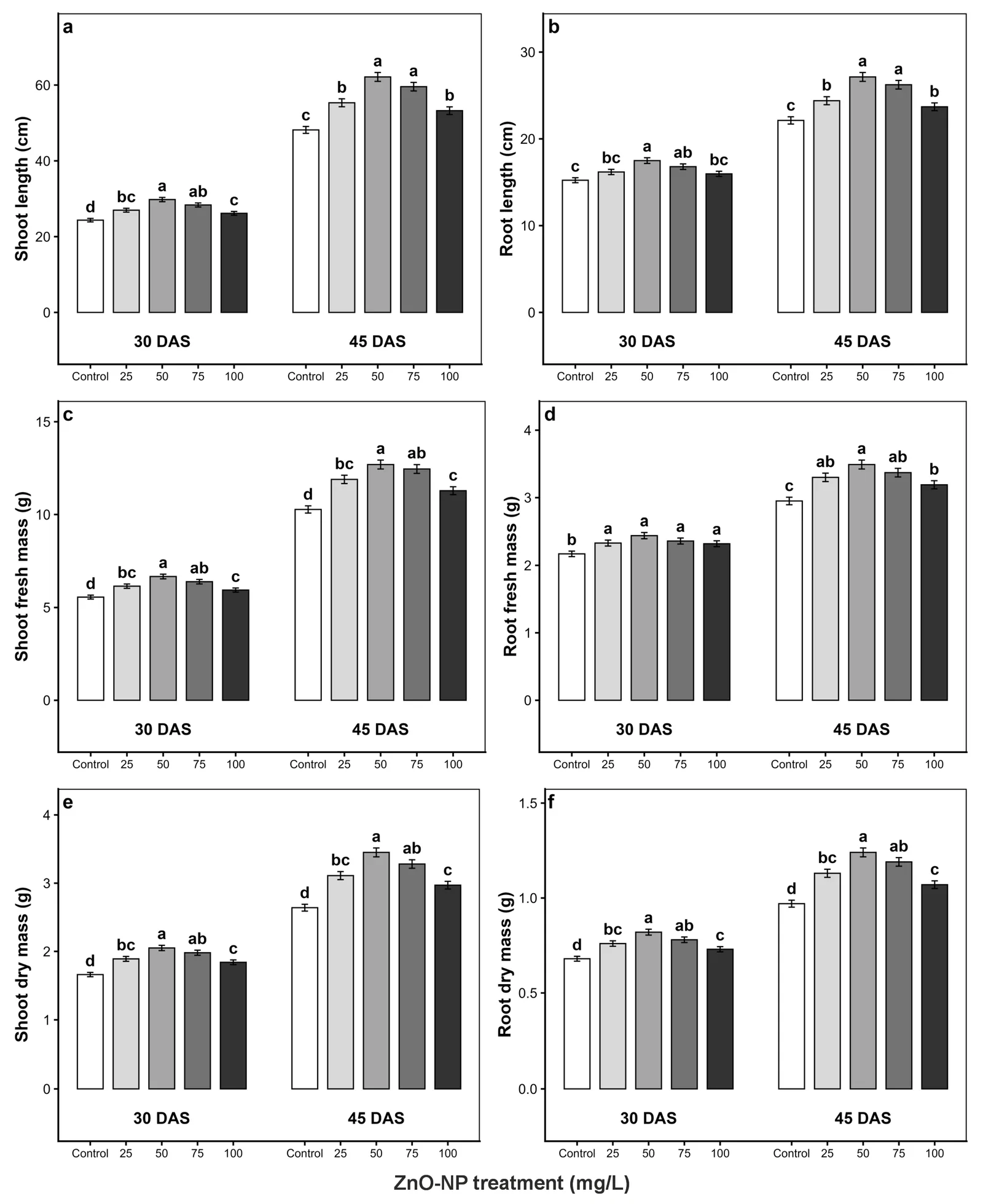
Parameteres of growth characteristics: (**a**) shoot length, (**b**) root length, (**c**) shoot fresh mass, (**d**) root fresh mass, (**e**) shoot dry mass and (**f**) root dry mass of *B. juncea* sprayed with different ZnO-NPs. All data are shown as mean ± SE. Significant difference between treatments on a particular day of treatment was determined by one-way ANOVA followed by Duncan’s post hoc test. Different letters above the error bar indicate statistical significance at *p* < 0.05.

**Figure 5:**
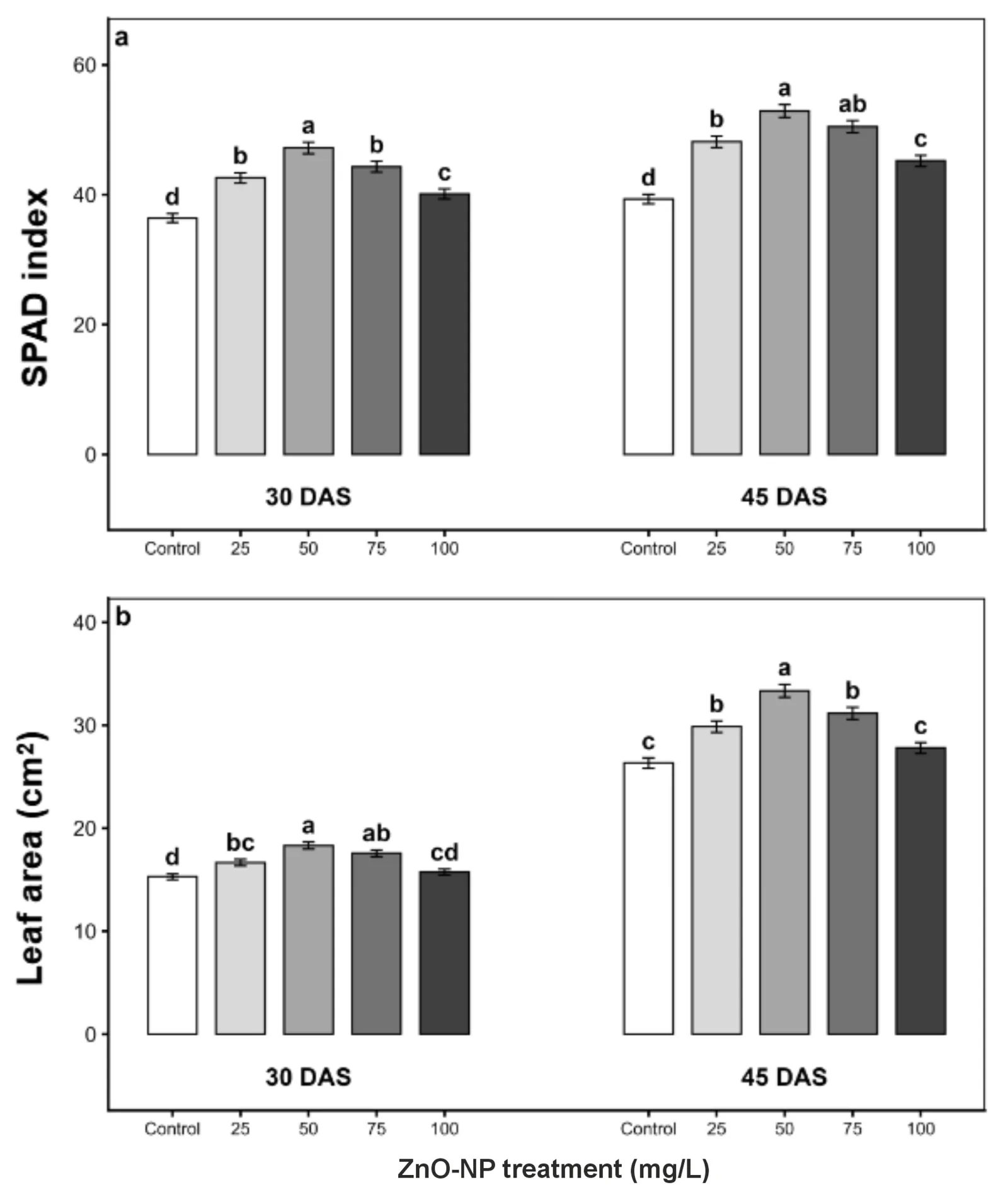
(**a**) SPAD index and (**b**) leaf area of *B. juncea* at 30 and 45 DAS in response to foliar ZnO-NP application. Data are mean ± SE (*n* = 4). Within each sampling date, different letters indicate significant differences among treatments according to Duncan’s multiple-range test at *p* ≤ 0.05.

Growth parameters such as shoot and root length, fresh and dry mass of the shoot and root, and leaf area were studied and analyzed. ZnO-NP treatment significantly influenced the above growth characteristics of *B. juncea* in a concentration-dependent manner. Among the treatments, the foliar spray of ZnO-NPs at 50 mg/L exhibited the maximum increase in shoot length, root length (Figure 3 and Figure 4), biomass accumulation, and leaf area (Figure 4 and Figure 5). ZnO-NPs at 50 mg/L enhanced the shoot length by 22.4% and 29%, root length by 14.8% and 22.6%, shoot fresh mass by 20% and 23.5%, root fresh mass by 12.4% and 18.3%, shoot dry mass by 23.5% and 30.6%, root dry mass by 20.6% and 27.8% respectively at 30 and 45 DAS against their respective control (Figure 4a–f).

### 2.3. SPAD Index (Chlorophyll Content) and Leaf Area

Foliar application of ZnO-NPs significantly enhanced chlorophyll content and leaf area in *B. juncea*, indicating possible improvement in photosynthetic efficiency. Among the treatments, ZnO-NPs at 50 mg/L recorded the maximum increases of about 29.6% and 34.4% (in chlorophyll content) and 19.8% and 26.6% (in leaf area) at 30 and 45 DAS, respectively, compared to their control (Figure 5a,b).

### 2.4. Stomatal Morphological Analysis

**Figure 6:**
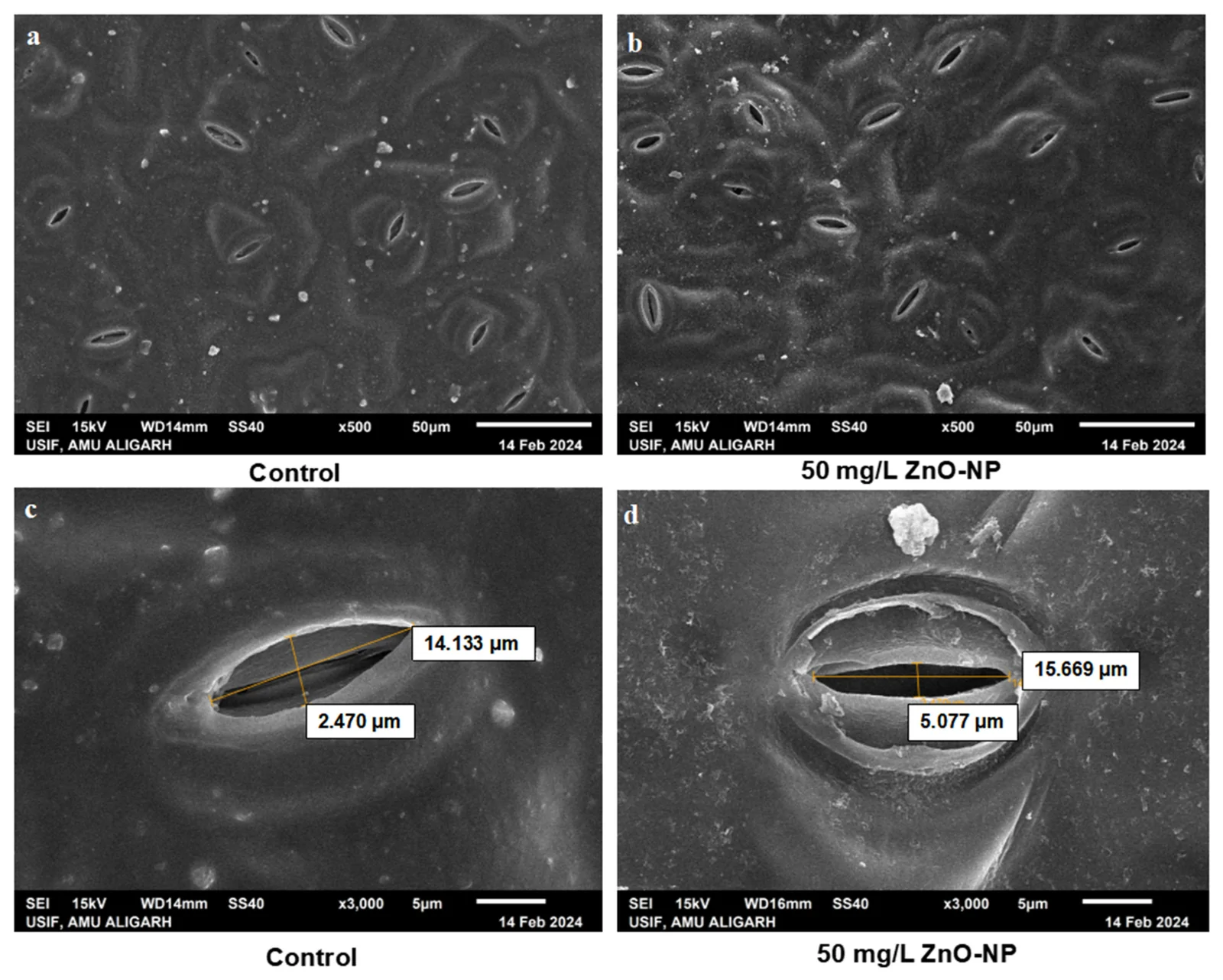
Representative SEM micrographs of leaf stomata of control (**a**,**c**) plants and plants treated with 50 mg/L ZnO-NPs (**b**,**d**) at 45 DAS. Images were obtained at 500× magnification (scale bar = 50 µm) and 3000× magnification (scale bar = 5 µm). Yellow lines indicate the measured stomatal pore dimension.

Stomatal morphology was compared between control plants and plants treated with 50 mg/L ZnO-NPs at 45 DAS. The stomatal aperture measured 2.47 µm in control plants and increased to 5.077 µm following treatment with 50 mg/L ZnO-NPs, corresponding to an increase of approximately 105.54% relative to the control (Figure 6). These observations indicate that the 50 mg/L treatment was associated with a wider stomatal aperture under the present experimental conditions.

### 2.5. Photosynthetic Attributes

**Figure 7:**
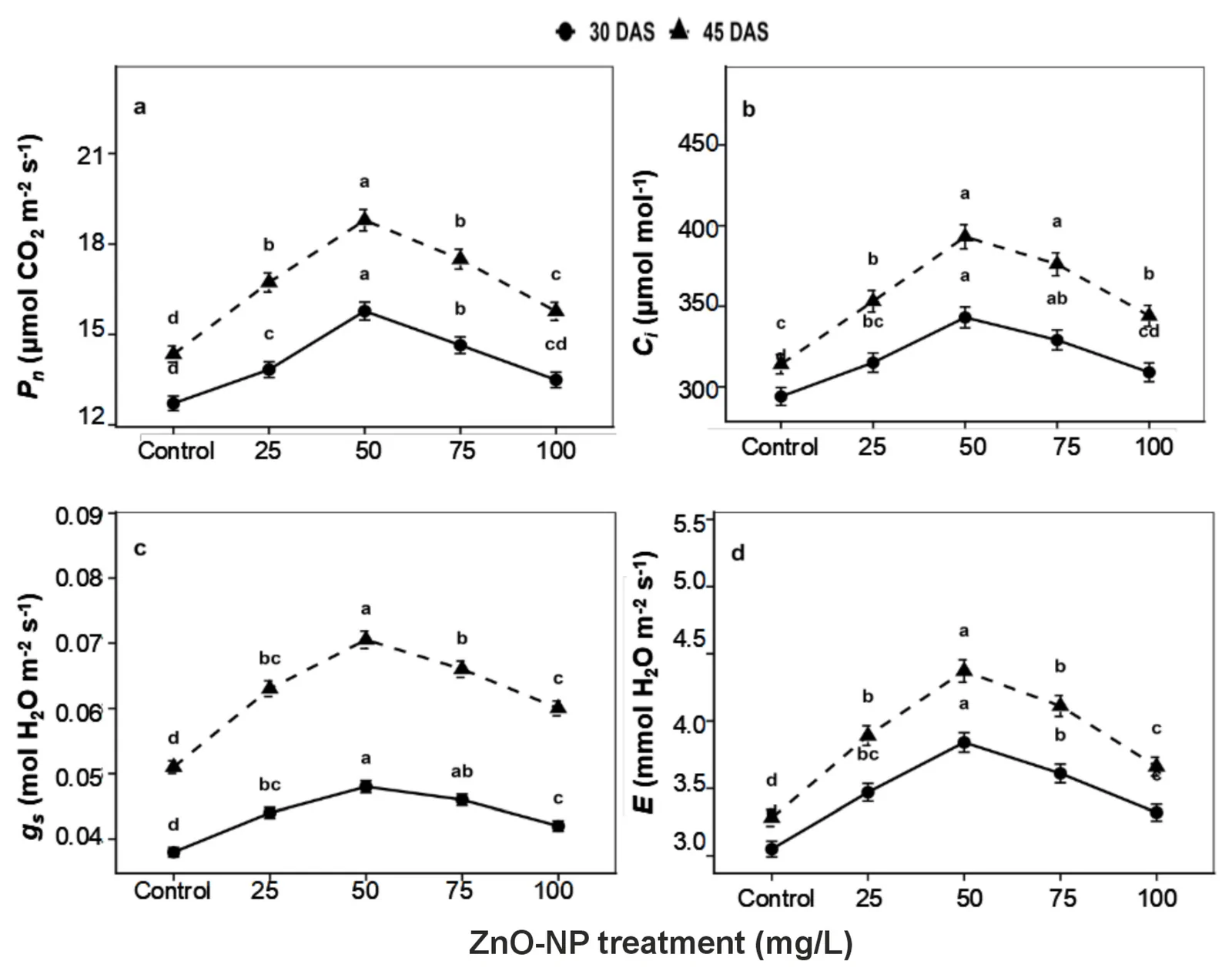
Effect of different concentrations of the ZnO-NPs applied through foliar mode on (**a**) net photosynthetic rate (P_N_), (**b**) internal CO_2_ concentration (Ci), (**c**) stomatal conductance (g_s_) and (**d**) transpiration rate (E) at 30 and 45 DAS of *B. juncea*. All data are shown as mean ± SE. Significant difference between treatments on a particular day of treatment was determined by one-way ANOVA followed by Duncan’s post hoc test. Different letters above the error bar indicate statistical significance at *p* < 0.05.

A similar pattern was observed for photosynthetic performance and associated gas-exchange attributes. Among all treatments, 50 mg/L of ZnO-NPs produced the most pronounced enhancement in net photosynthetic rate (P_N_), intercellular CO_2_ concentration (C_i_), stomatal conductance (g_s_), and transpiration rate (E) (Figure 7). At 30 DAS, these parameters increased by 24.07%, 16.6%, 26.31%, and 25.9%, respectively, whereas at 45 DAS, the corresponding increases were 31%, 25.16%, 38.2%, and 33.2%, respectively, compared to their respective controls. However, the positive responses declined marginally at elevated ZnO-NP concentrations (75–100 mg/L), suggesting the onset of concentration-dependent inhibitory effects at higher application levels.

### 2.6. Chlorophyll Fluorescence Analysis

**Figure 8:**
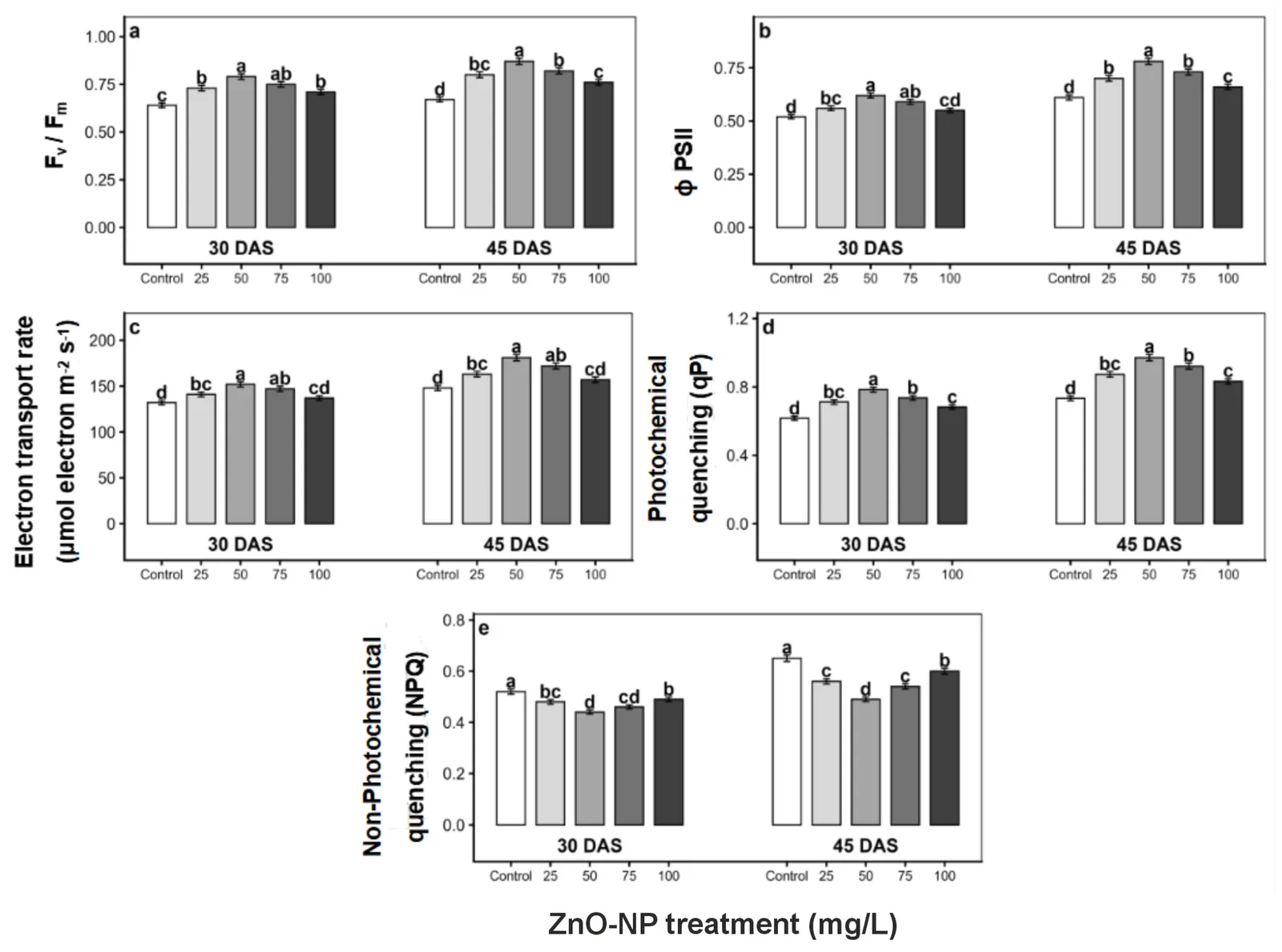
Effect of different concentrations of the ZnO-NPs applied through foliar mode on chlorophyll fluorescence analysis: (**a**) maximum quantum yield of PSII (Fv/Fm), (**b**) actual PSII efficiency (ΦPSII), (**c**) electron transport rate (ETR), (**d**) photochemical quenching (qP) and (**e**) non-photochemical quenching (NPQ) at 30 and 45 DAS of *B. juncea.* All data are shown as mean ± SE. Significant difference between treatments on a particular day of treatment was determined by one-way ANOVA followed by Duncan’s post hoc test. Different letters above the error bar indicate statistical significance at *p* < 0.05.

A distinct concentration-dependent effect of ZnO-NPs was evident in the chlorophyll fluorescence characteristics of mustard plants. The 50 mg/L treatment proved to be the most effective, resulting in the greatest enhancement of PSII maximum quantum efficiency (Fv/Fm), effective quantum yield (ΦPSII), electron transport rate (ETR), and photochemical quenching (qP), together with a marked reduction in non-photochemical quenching (NPQ) (Figure 8). Relative to the control, Fv/Fm, ΦPSII, and qP increased by 23.72%, 19.23%, and 27.06%, respectively, at 30 DAS, while corresponding improvements of 28.78%, 28.0% and 32.46% were recorded at 45 DAS. Likewise, ETR reached its highest value with the 50 mg/L treatment, showing a 22.3% increase at 45 DAS. Conversely, NPQ decreased by 15.38% and 24.61% at 30 and 45 DAS, respectively. Nevertheless, exposure to higher concentrations (75–100 mg/L) resulted in declines in Fv/Fm, ΦPSII, ETR, and qP, along with an increase in NPQ.

### 2.7. Carbohydrate and Sugar Contents

**Figure 9:**
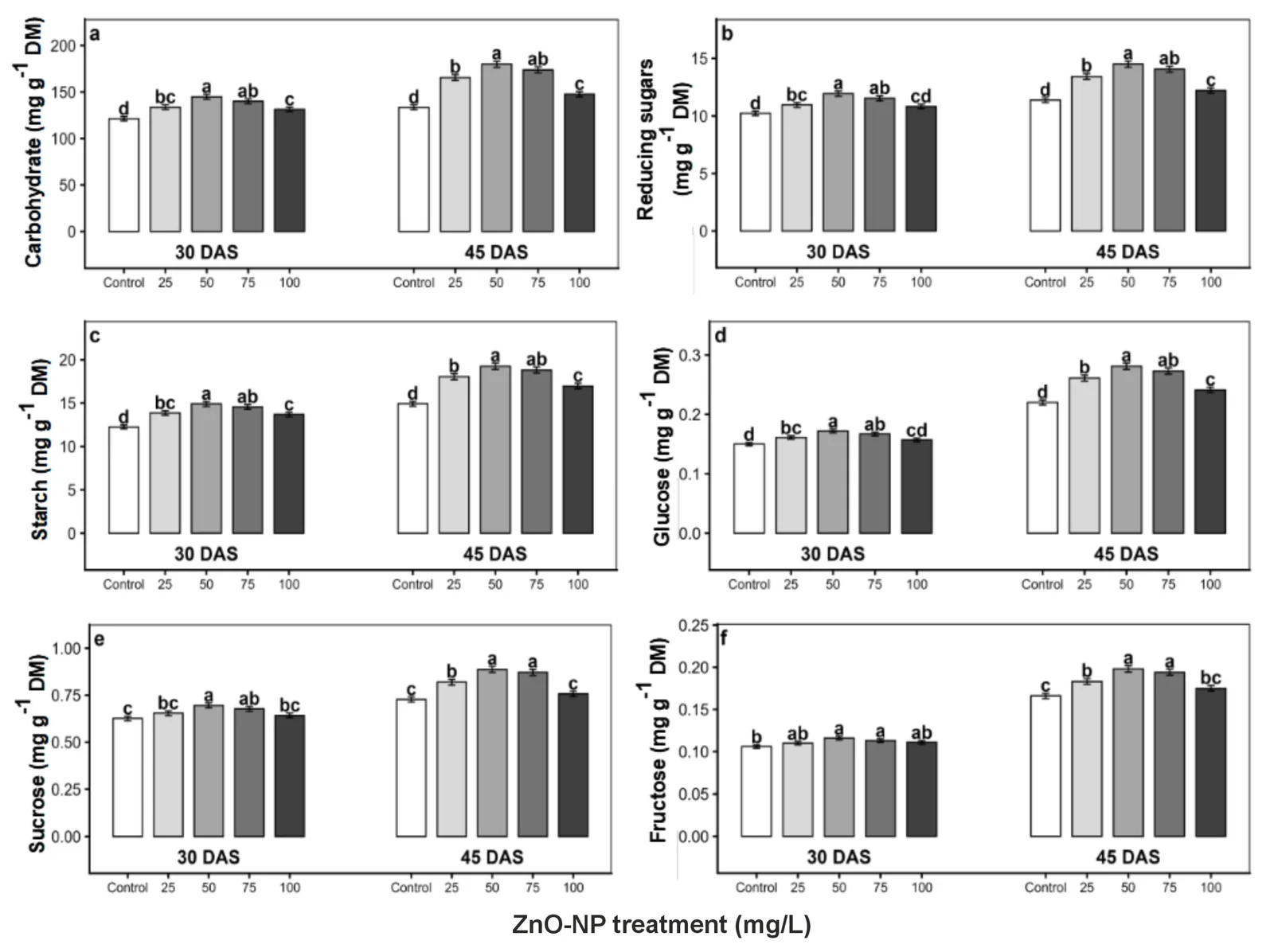
Effect of different concentrations of the ZnO-NPs applied through foliar mode on the contents of (**a**) carbohydrate, (**b**) reducing sugar, (**c**) starch, (**d**) glucose, (**e**) sucrose and (**f**) fructose at 30 and 45 DAS of *B. juncea*. All data are shown as mean ± SE. Significant difference between treatments on a particular day of treatment was determined by one-way ANOVA followed by Duncan’s post hoc test. Different letters above the error bar indicate statistical significance at *p* < 0.05.

The accumulation of carbohydrates and soluble sugars responded positively to ZnO-NP application, with the magnitude of the response varying according to NP concentration. Among all treatments, 50 mg/L ZnO-NPs resulted in the greatest enhancement in total carbohydrates, reducing sugars, starch, glucose, sucrose, and fructose contents (Figure 9). Compared to the control, total carbohydrate content increased by 19.43% and 34.8% at 30 and 45 DAS, respectively, and reducing sugars by 16.71% and 27.5%, and starch by 21.8% and 29.0% at 30 and 45 DAS, respectively. Likewise, glucose rose by 14.67% and 27.7% at 30 and 45 DAS, respectively, and sucrose by 11.0% and 22.0%, and fructose by 9.43% and 19.3% at 30 and 45 DAS, respectively. However, the stimulatory effects were less pronounced at higher concentrations (75–100 mg/L).

### 2.8. Protein Estimation

**Figure 10:**
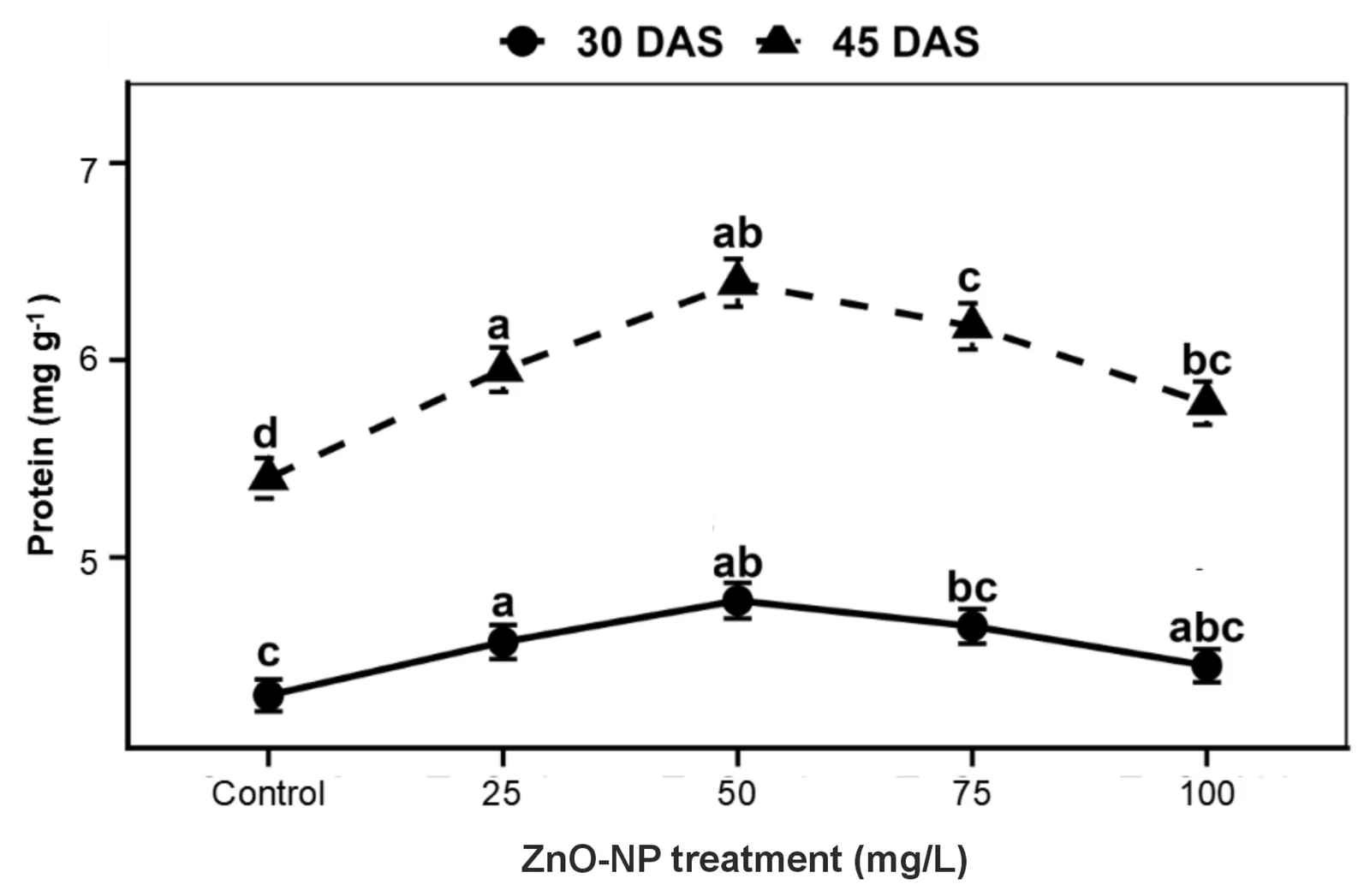
Effect of different concentrations of the ZnO-NPs applied through foliar application on the protein content in the leaves of *B. juncea* at 30 and 45 DAS. All data are shown as mean ± SE. Significant difference between treatments on a particular day of treatment was determined by one-way ANOVA followed by Duncan’s post hoc test. Different letters above the error bar indicate statistical significance at *p* < 0.05.

Foliar application of ZnO-NPs significantly increased protein content, with the greatest response observed at 50 mg/L. Under this treatment, protein levels increased by 11.16% and 18.3% at 30 and 45 DAS, respectively, compared with the control plants (Figure 10).

### 2.9. Biochemical and Antioxidative Enzyme Analysis

**Figure 11:**
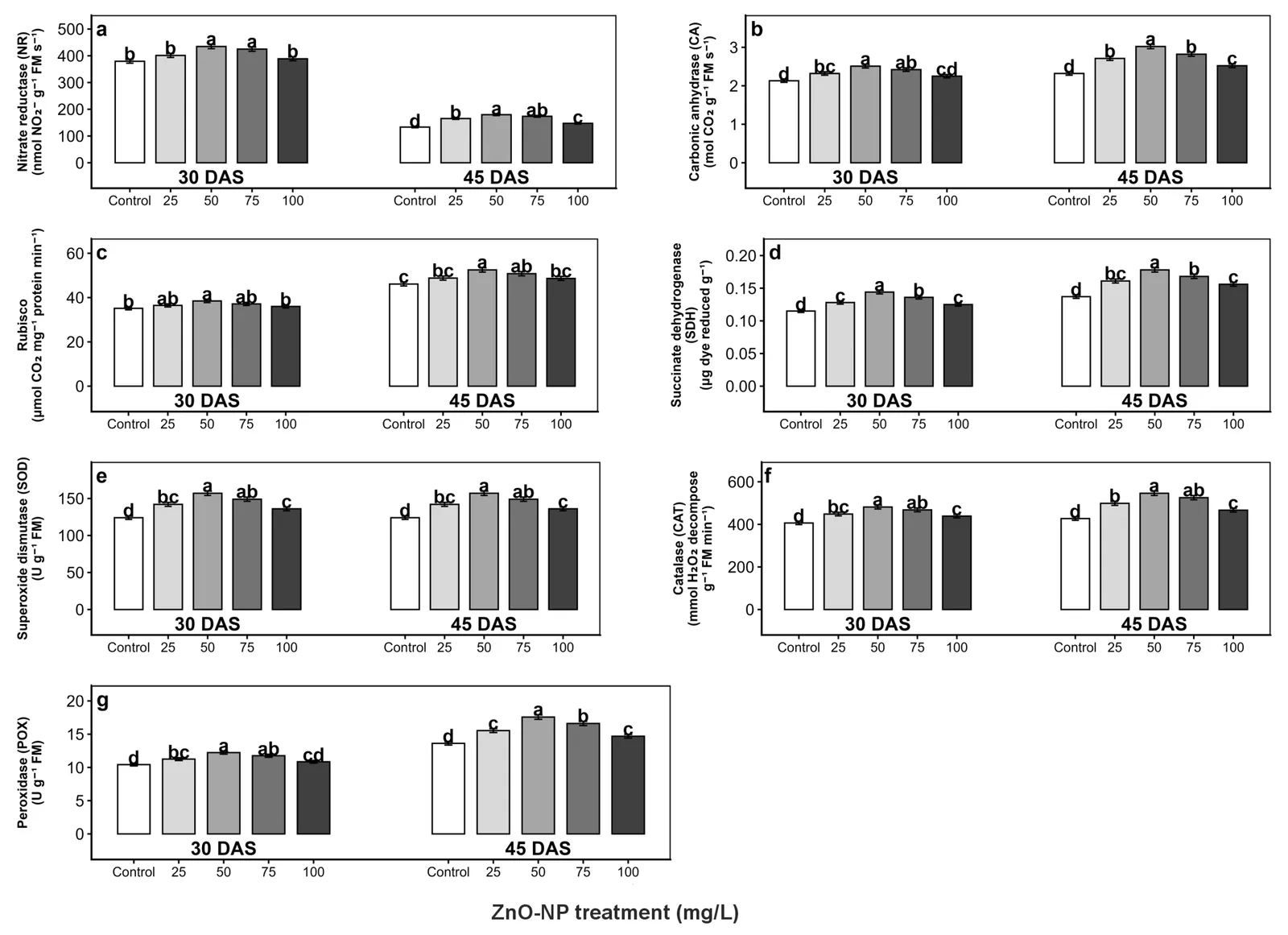
Effect of different concentrations of the ZnO-NPs applied through foliar application on the activities of (**a**) nitrate reductase, (**b**) carbonic anhydrase, (**c**) Rubisco, (**d**) succinate dehydrogenase, (**e**) superoxide dismutase, (**f**) catalase and (**g**) peroxidase in the leaves of *B. juncea* at 30 and 45 DAS. All data are shown as mean ± SE. Significant difference between treatments on a particular day of treatment was determined by one-way ANOVA followed by Duncan’s post hoc test. Different letters above the error bar indicate statistical significance at *p* < 0.05.

A similar concentration-dependent response was observed for nitrogen metabolism, photosynthetic, and antioxidant enzyme activities following ZnO-NP application. Among all treatments, 50 mg/L ZnO-NPs produced the greatest enhancement in nitrate reductase (NR), carbonic anhydrase (CA), ribulose-1,5-bisphosphate carboxylase/oxygenase (Rubisco), succinate dehydrogenase (SDH), superoxide dismutase (SOD), catalase (CAT), and peroxidase (POX) activities (Figure 11). Compared with the control, NR activity increased by 14.54% and 25.7% at 30 and 45 DAS, respectively, while CA activity increased by 17.8% and 30.2%. Similarly, Rubisco activity increased by 9.5% and 13.9% at 30 and 45 DAS, respectively, whereas SDH activity increased by 25.21% and 29.9% at 30 and 45 DAS, respectively. The antioxidant defense system also responded positively to ZnO-NP treatment, with SOD activity increasing by 26.6% and 34.3%, CAT activity by 18.4% and 27.8%, and POX activity by 17.37% and 29.0% at 30 and 45 DAS, respectively. However, the beneficial effects were reduced at higher ZnO-NP concentrations (75–100 mg/L).

### 2.10. Proline and Glutathione Contents

**Figure 12:**
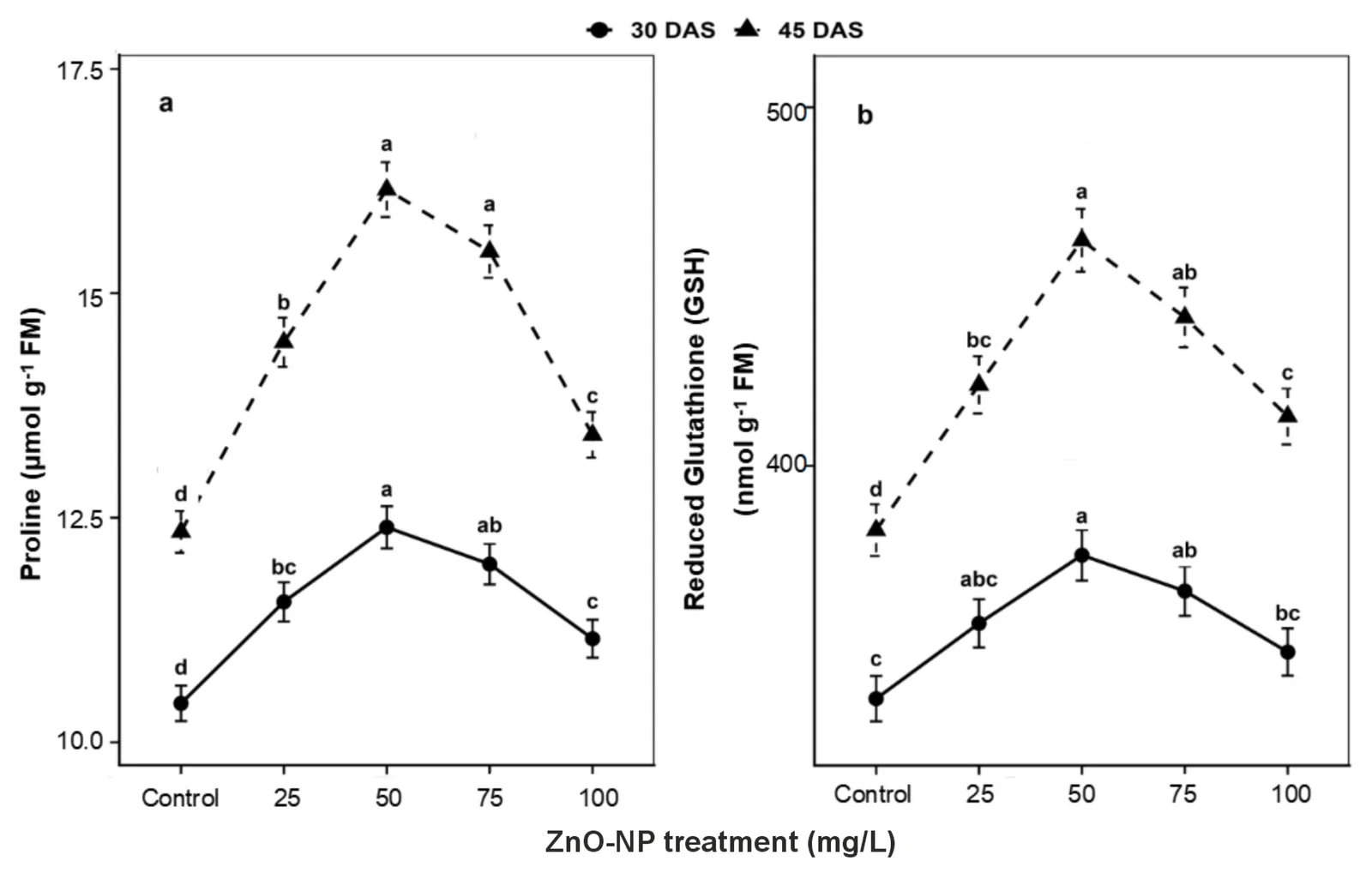
Effect of different concentrations of the ZnO-NPs applied through foliar application on the contents of (**a**) proline and (**b**) reduced glutathione in the leaves of *B. juncea* at 30 and 45 DAS. All data are shown as mean ± SE. Significant difference between treatments on a particular day of treatment was determined by one-way ANOVA followed by Duncan’s post hoc test. Different letters above the error bar indicate statistical significance at *p* < 0.05.

A comparable response was observed in the accumulation of proline and non-enzymatic antioxidant compounds following the application of ZnO-NPs. Among all treatments, 50 mg/L ZnO-NPs resulted in the greatest increase in proline and glutathione (GSH) contents (Figure 12a,b). Relative to the control, proline concentration increased by 18.79% and 31.0% at 30 and 45 DAS, respectively, while GSH content increased by 12.0% and 21.15% at 30 and 45 DAS, respectively.

### 2.11. Quantification and Histochemical Localization of Reactive Oxygen Species

**Figure 13:**
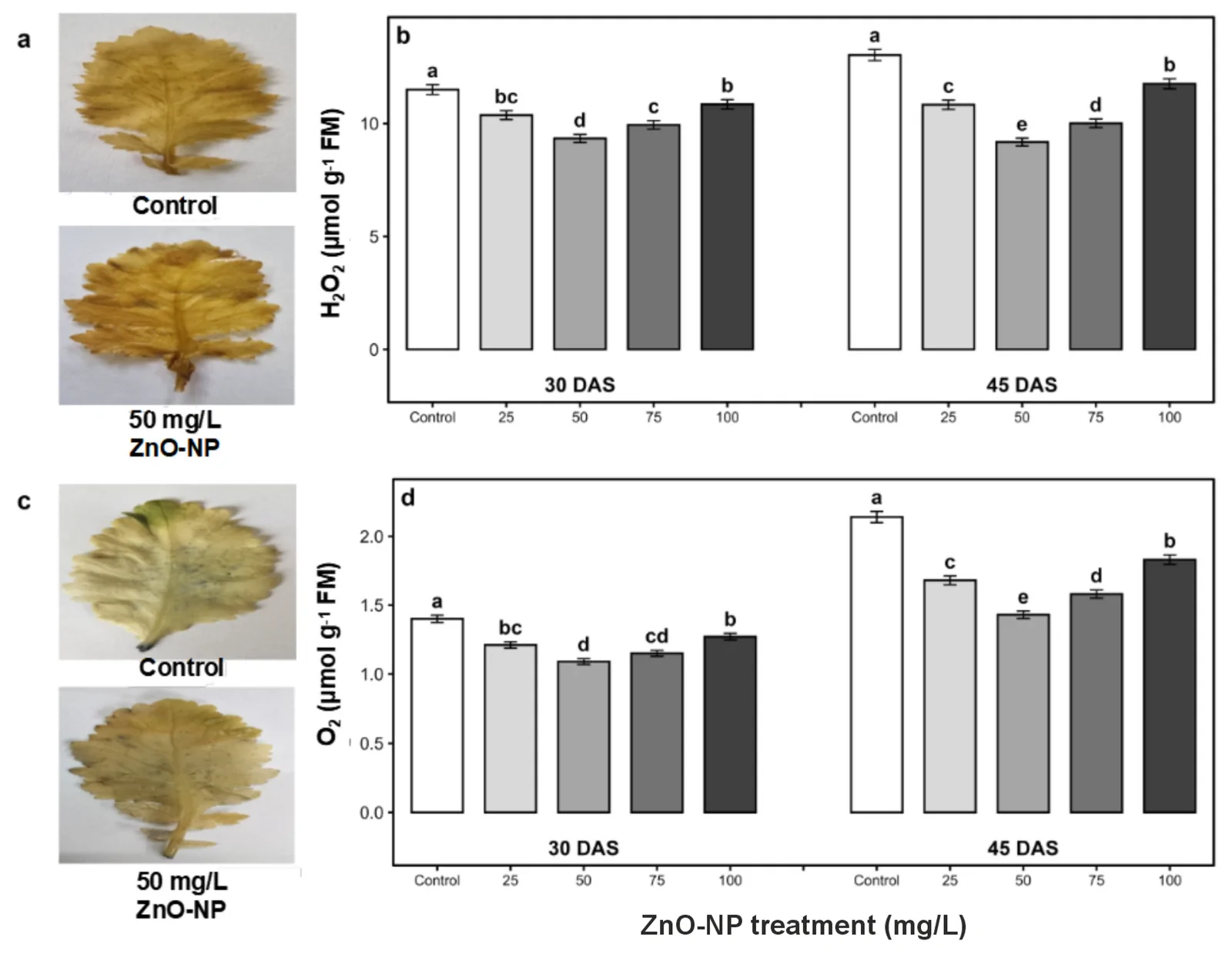
Effect of different concentrations of the ZnO-NPs applied through foliar application on the leaf histochemical localization by 3,3- diaminobenzidine (at 45 DAS) and the content (at 30 and 45 DAS) of H_2_O_2_ (**a**,**b**) and the leaf histochemical localization by nitro blue tetrazolium (NBT) (at 45 DAS) and the content (at 30 and 45 DAS) of O_2_•^−^ (**c**,**d**) in *B. juncea*. Data are mean ± SE (*n* = 4). Within each sampling date, different letters indicate significant differences at *p* ≤ 0.05.

A marked reduction in ROS was recorded in response to ZnO-NP treatment. The 50 mg/L concentration proved most effective in reducing the accumulation of hydrogen peroxide (H_2_O_2_) and superoxide radicals (O_2_^•−^). Relative to the control, H_2_O_2_ and O_2_^•−^ contents decreased by 18.8% and 22.14%, respectively, at 30 DAS. At 45 DAS, the corresponding reductions reached 29.5% and 33.2%, respectively, with the observed reductions being more pronounced at 45 DAS than at 30 DAS (Figure 13a,b). Nevertheless, the ROS-lowering effect weakened at higher ZnO-NP concentrations (75–100 mg/L).

Histochemical detection of H_2_O_2_ and O_2_^•−^ in leaf tissues further corroborated the biochemical findings. For H_2_O_2_ localization, control leaves exhibited extensive dark brown staining, indicating greater peroxide accumulation. In contrast, leaves treated with 50 mg/L of ZnO-NPs displayed the faintest and least abundant brown spots (Figure 13a). Similarly, the accumulation of O_2_^•−^, visualized as blue staining, was most intense in control leaves but was markedly reduced in plants receiving foliar application of 50 mg/L of ZnO-NPs (Figure 13b).

**Figure 14:**
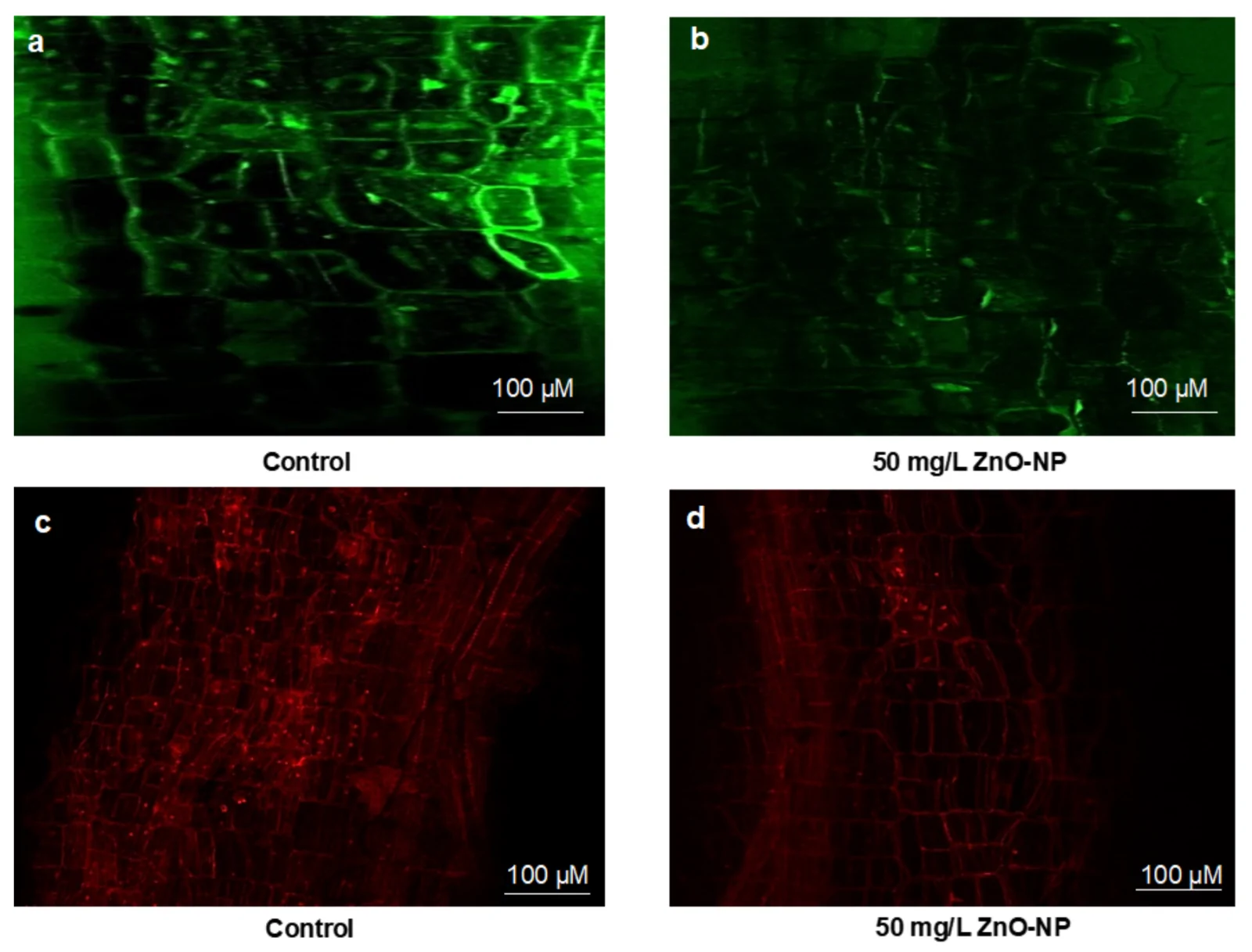
Confocal microscopic analysis of ROS accumulation and cell viability in roots of 45-day-old *B. juncea* plants. (**a**,**b**) ROS localization in control (**a**) and 50 mg/L ZnO-NP-treated (**b**) roots using DCFH-DA staining (excitation: 488 nm; emission: 520–530 nm); stronger green fluorescence indicates greater ROS accumulation. (**c**,**d**) Cell viability analysis in control (**c**) and 50 mg/L ZnO-NP-treated (**d**) roots using propidium iodide staining (excitation: 535–543 nm; emission: 617–620 nm); stronger red nuclear fluorescence indicates greater membrane damage and lower cell viability.

In addition, confocal microscopy revealed the lowest fluorescence intensity in the roots of plants treated with 50 mg/L ZnO-NPs, further confirming reduced ROS accumulation compared with untreated control plants (Figure 14a,b).

### 2.12. Confocal Microscopy for Cell Viability

Propidium iodide (PI) staining was employed to assess root cell death. PI selectively enters cells with damaged membranes and binds to DNA, producing red fluorescence in non-viable cells, whereas living cells with intact membranes exclude the dye and remain unstained. Control roots exhibited strong red fluorescence, reflecting a higher degree of membrane permeability and cellular damage (Figure 14c). Conversely, roots exposed to 50 mg/L ZnO-NPs showed considerably weaker fluorescence signals and a more organized cellular architecture, indicating preservation of membrane integrity and improved cell survival (Figure 14d).

### 2.13. Estimation and Histochemical Detection of Lipid Peroxidation

**Figure 15:**
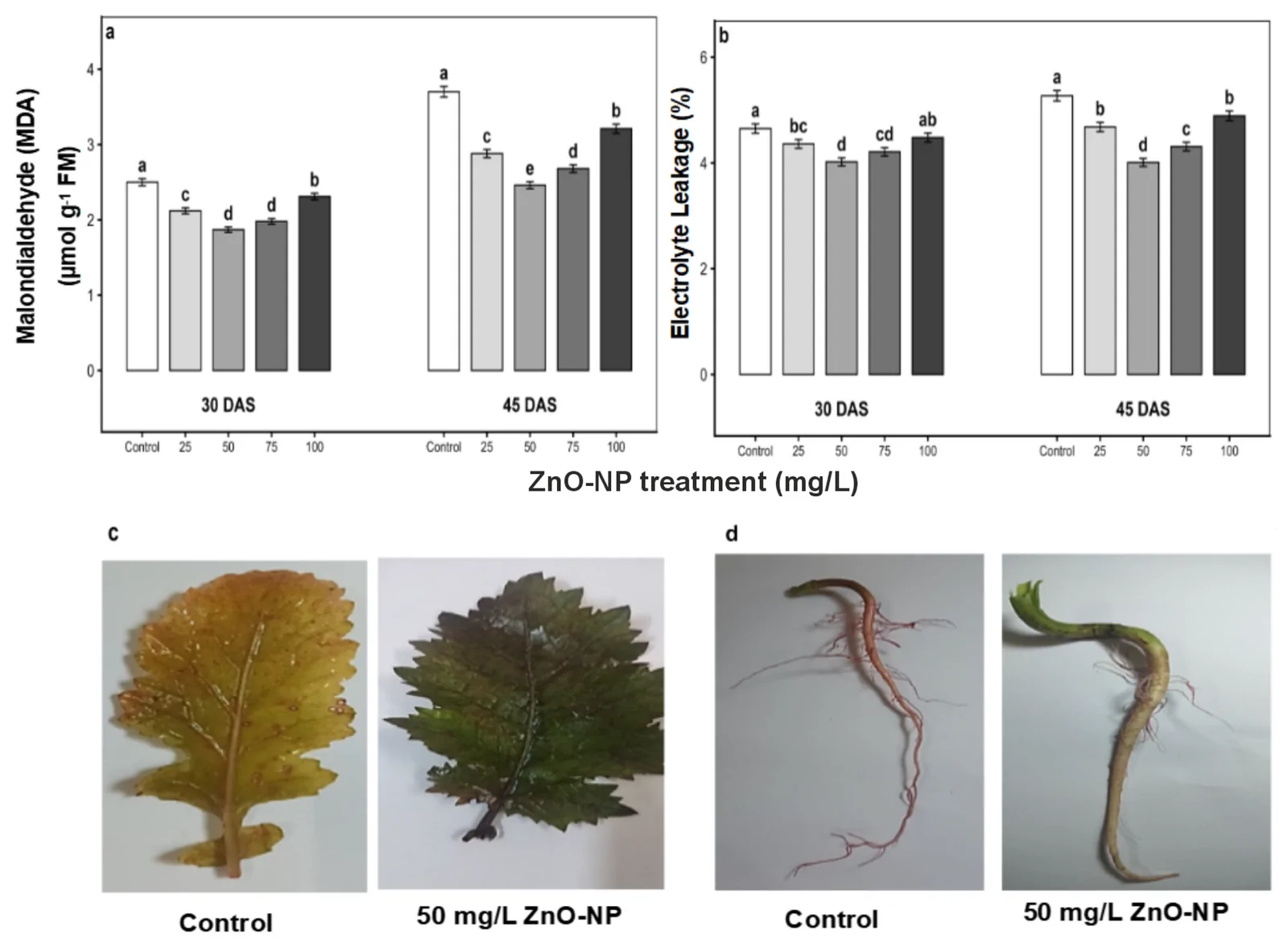
Effect of different concentrations of the ZnO-NPs applied through foliar application on plant membrane damage and stability. (**a**) Malondialdehyde (MDA) content and (**b**) electrolyte leakage (EL) at 30 and 45 DAS of *B. juncea*. Histochemical detection of lipid peroxidation with Schiff’s reagent (**c**) in the leaves and (**d**) in the roots of 45-day-old *B. juncea*. All data are presented as mean ± SE. Significant differences among treatments on a particular day were determined by one-way ANOVA followed by Duncan’s multiple-range test. Different letters above the bars indicate significant differences at *p* < 0.05.

The application of ZnO-NPs, particularly at 50 mg/L, markedly improved membrane stability and reduced indicators of oxidative damage. Malondialdehyde (MDA), a commonly used marker of lipid peroxidation, decreased by 25.2% and 33.5% at 30 and 45 DAS, respectively, following foliar application of 50 mg/L ZnO-NPs. Similarly, electrolyte leakage (EL), an indicator of membrane permeability, decreased by 13.54% and 23.9% at 30 and 45 DAS, respectively, indicating improved membrane integrity (Figure 15b).

These results were further validated through histochemical detection of lipid peroxidation in leaves (Figure 15c) and roots (Figure 15d). In this assay, deeper pink staining indicated higher levels of lipid peroxidation. The most intense pink coloration was observed in both leaves and roots of the control plants. In contrast, the weakest staining was observed in plants treated with 50 mg/L ZnO-NPs, indicating a marked reduction in lipid peroxidation.

### 2.14. Leaf Elemental Composition

Foliar application of ZnO-NPs significantly affected the elemental composition of *B. juncea* leaves at 45 DAS. Among the tested treatments, plants receiving 50 mg/L ZnO-NPs showed the highest relative abundance of the analyzed elements, including C, Mg, S, P, K, Ca, and Zn, as determined by SEM–EDX analysis. Relative to the control, the values for C, Mg, S, P, K, and Ca increased by 10.54%, 13.23%, 12.67%, 16.44%, 22.61%, and 18.64%, respectively. The Zn signal showed the largest relative increase, reaching approximately 33.3% above the control (Table 1).

### 2.15. Yield Parameters

**Figure 16:**
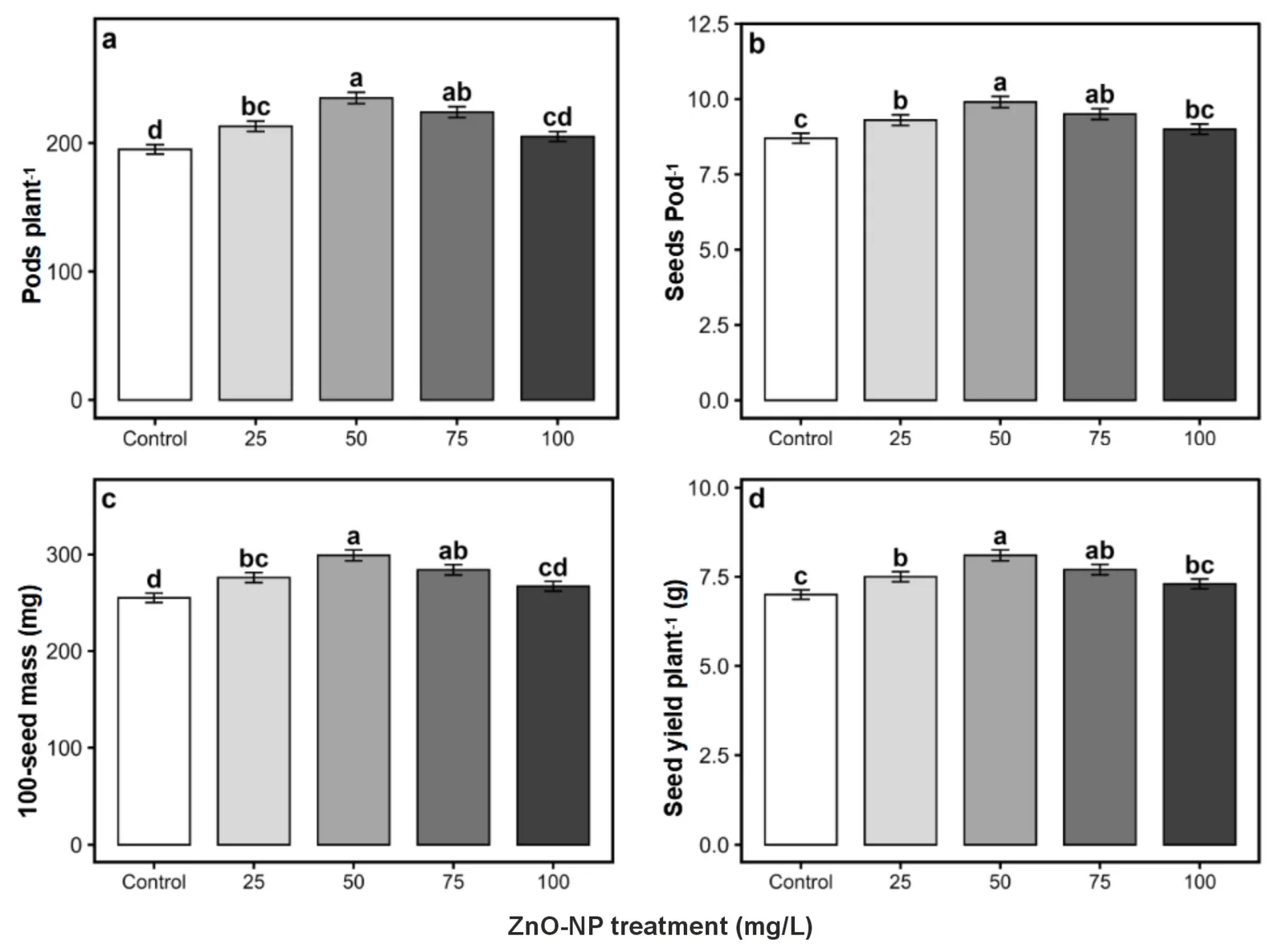
Effect of different concentrations of the ZnO-NPs applied through foliar application on yield attributes at 120 DAS of *B. juncea*. (**a**) Pods per plant, (**b**) seeds per pod, (**c**) 100-seed mass and (**d**) seed yield per plant. All data are represented as mean ± SE. Significant difference between treatments on a particular day of treatment was determined by one-way ANOVA followed by Duncan’s post hoc test. Different letters above the error bar indicate statistical significance at *p* < 0.05.

Yield-related attributes exhibited a marked improvement following the application of ZnO-NPs, with the 50 mg/L treatment producing the most pronounced response. Under this treatment, the number of pods per plant, seeds per pod, 100-seed weight, and seed yield per plant increased by 20.5%, 13.8%, 17.3% and 15.7%, respectively, compared to the control at 120 DAS of *B. juncea* (Figure 16).

## 3. Discussion

The application of nanotechnology in agriculture has emerged as a promising strategy for improving crop performance and resource-use efficiency. Nanoparticles have been reported to influence a wide range of physiological and biochemical processes, thereby contributing to enhanced plant growth and productivity [34,35]. Among the investigated nanomaterials, ZnO-NPs have received considerable attention because they can provide zinc in a bioavailable form and influence metabolic processes involved in plant growth and development [36]. In the present investigation, foliar supplementation with ZnO-NPs markedly influenced the growth, photosynthetic characteristics, biochemical constituents, and yield attributes of *B. juncea* var. *Varuna*. The responses varied with NP concentration, with the 50 mg/L treatment yielding the most favorable outcomes across most of the studied parameters. Collectively, these findings suggest that 50 mg/L represents the most favorable concentration among those tested for promoting photosynthetic performance, whereas higher concentrations progressively reduce the beneficial response and may indicate the onset of phytotoxic effects.

The various characterizations confirmed the ZnO-NPs with desirable structural and morphological properties. XRD analysis revealed distinct diffraction peaks that closely matched the standard ZnO pattern, i.e., JCPDS card no. 36-1451, confirming the formation of a highly crystalline ZnO phase [37]. FTIR spectroscopy further verified the chemical characteristics of the NPs. SEM analysis revealed that the NPs exhibited an irregular, aggregated morphology. Such agglomeration is commonly observed in metal oxide NPs due to their high surface energy and strong interparticle attractions [38]. The observed structural features, including high crystallinity, low lattice distortion, nanoscale crystallite size, and surface functional groups, are important determinants of NP’s reactivity, stability, and biological interactions, thereby influencing their effectiveness in agricultural and plant-related applications.

The stimulatory effects of ZnO-NPs on plant growth at lower concentrations may be linked to Zn’s fundamental role in regulating several metabolic and developmental processes. Zinc is an important micronutrient that facilitates enzyme function, hormone formation, chlorophyll formation and metabolism of nutrients for plant growth. The optimal growth at 50 mg/L implies an optimal ratio between Zn availability and plant needs. The attenuated growth at 100 mg/L might be a result of physiological limitations that come as a consequence of increased nanoparticle concentrations. Since there was no control using bulk ZnO and Zn concentration, the impact of nanoparticle-specific effects could not be separated from the Zn effects [28,39].

The application of ZnO-NPs is widely attributed to the regulation of photosynthesis via photosynthetic pigments, with effects that depend on concentration: low concentrations enhance chlorophyll synthesis, whereas high concentrations cause oxidative stress that prevents accumulation [40,41]. In plant cells, ZnO-NPs can interact with molecules within chloroplasts, affecting the movement and metabolism of chlorophyll. The zinc content is vital for the production of protochlorophyllide, chloroplast formation, and the activation of photosynthetic enzymes, thereby increasing photosynthetic activity [41,42] and enhancing chlorophyll production. Chlorophyll content is significant in determining the rate of photosynthesis in the plant. In the present study, the SPAD index increased markedly at low-to-moderate ZnO-NP concentrations, with the highest value recorded at 50 mg/L, whereas the response declined at 100 mg/L. This pattern is consistent with a concentration-dependent effect of ZnO-NPs on chlorophyll status and photosynthetic performance. The dose-dependent effects of ZnO-NPs on chlorophyll content have been noticed among other organisms as well; for instance, 50 mg/L of ZnO-NPs increased the chlorophyll content in *Pisum sativum* by 42–51%, and 120 mg/pot or ~100 ppm ZnO-NPs as soil application increased chlorophyll content by 10–24.6% in *Triticum aestivum* [22,40]. Meanwhile, the reduction in SPAD index with increasing ZnO-NP levels (100 mg/L) reflects chlorophyll loss from the leaves because of the impact of ZnO-NPs, which causes oxidation stress due to the high concentrations of the nanoparticles, leading to alterations in chloroplasts and a reduction in granum/thylakoids [41]. The degradation of chlorophyll in this case can be attributed to alterations in the pigment–protein complex, increased activity of the chlorophyllase enzyme, and inhibition of photosynthetic pigment biosynthesis due to exposure to high concentrations of NPs [43,44]. Thus, previous studies have reported a biphasic effect of ZnO-NPs, in which low concentrations increase chlorophyll content, whereas high concentrations reduce it [28,39,45].

The improved photosynthetic activity recorded in ZnO-NP-treated plants could be related to enhanced Zn availability and its contribution to the proper functioning of photosynthetic processes. Nevertheless, the present investigation did not examine the molecular or protein-level components governing chlorophyll formation, Rubisco regulation or stomatal signaling. Thus, the physiological improvements observed here should not be considered direct evidence of nanoparticle-induced regulation of any particular molecular pathway. Furthermore, the lack of a bulk ZnO treatment prevents a clear distinction between responses arising from the nanoscale characteristics of ZnO-NPs and those resulting from Zn nutrition. Future investigations using appropriate bulk ZnO and soluble Zn controls, combined with molecular, biochemical and proteomic approaches, would help clarify the mechanisms responsible for the physiological responses observed following ZnO-NP treatment. The enhanced SDH activity may reflect increased respiratory metabolism and energy availability. In contrast, the weaker photosynthetic response observed at 75–100 mg/L suggests that higher ZnO-NP concentrations may impose physiological constraints. Previous studies have associated excessive ZnO-NP exposure with oxidative stress, altered stomatal regulation, disruption of chloroplast structure, reduced photosynthetic pigment content, and inhibition of photosynthetic enzymes [44,45]. Similar concentration-dependent responses involving antioxidant metabolism, stomatal function, and photosynthetic performance have also been reported in other plant species [46,47,48,49]. Molecular mechanisms proposed in previous studies include changes in the regulation of genes associated with Rubisco, the ascorbate–glutathione (AsA–GSH) antioxidant system, and plasma membrane H^+^-ATPase activity [50]. However, these molecular mechanisms were not directly examined in the present study. Therefore, the current results support an association between the 50 mg/L ZnO-NP treatment and improved stomatal and photosynthetic performance, but do not establish the underlying molecular mechanism.

The foliar application of ZnO-NPs played a crucial role in regulating oxidative metabolism and improving the antioxidant defense mechanism in *B. juncea*. ROS, such as H_2_O_2_ and O_2_^•−^, are produced throughout the cell’s physiological activities, but the overproduction of these causes oxidative stress and damage to cell membranes and metabolism. There was a significant decrease in H_2_O_2_ and O_2_^•−^ levels, as evidenced biochemically and by staining. The decrease in brown and blue staining in the leaves and in fluorescence in the roots indicated that oxidative stress was inhibited by ZnO-NPs [50].

It has been found that NPs may affect the cellular redox balance, which depends on their concentration, physicochemical properties, and interactions with antioxidant systems [51]. Under favorable concentrations, ZnO-NPs can act as regulators of ROS, activating antioxidant pathways and redox signaling [52]. Zinc ions are micronutrients that play a major role in the stabilization of biomembranes, protein synthesis, and the activation of metalloenzymes involved in antioxidative metabolism. Thus, the lower ROS content observed in ZnO-NP-treated plants may be associated with enhanced antioxidant enzyme activity. Similar findings have been reported in tomato (*Solanum lycopersicum* L.), where ZnO-NP application reduced ROS accumulation [47]. Dimkpa et al. [27] also reported improved redox balance following the application of Zn-based nanoparticles.

Histochemical localization analysis further supported the biochemical findings. In control plants, there was strong dark brown and blue staining, indicating high accumulation of H_2_O_2_ and O_2_^•−^ ions, whereas in ZnO-NP-treated plants, staining was quite weak. A reduction in ROS content appears to be strongly correlated with enhanced activity of antioxidative enzymes, such as SOD, CAT, and POX. SOD acts as the first-line defense mechanism, converting superoxide into H_2_O_2_, which is further converted into water and oxygen by CAT and POX. Thus, the increased enzyme activity suggests that a highly efficient ROS-scavenging mechanism was activated, protecting against oxidative damage. Enhanced activity of antioxidant enzymes upon treatment with ZnO-NPs has also been observed in wheat, rice, and *Arabidopsis thaliana* [26,53].

Apart from antioxidant protection, another function attributed to ZnO-NPs is the stimulation of enzyme activities associated with nitrogen metabolism, photosynthesis, and respiration. Increased activities of NR and CA indicate improved nitrogen assimilation and carbon dioxide fixation. In contrast, increased activities of Rubisco and SDH indicate improved efficiency of photosynthetic carbon metabolism and mitochondrial respiration, respectively. Since zinc functions as a crucial cofactor in several enzymes and proteins, such as chlorophyll synthesis, ATP generation, and electron transport, the increased enzymatic activity may arise from higher zinc bioavailability and metabolic activation [26,53]. Improved photosynthetic activity and respiratory metabolism were likely responsible for increased energy availability and enhanced repair of cellular components [47].

Lower MDA content and electrolyte leakage (EL) levels supported the effectiveness of ZnO-NPs as protectors against oxidative damage to plant membranes. An increase in ROS concentration usually triggers peroxidation of membrane lipids, leading to increased MDA levels and higher membrane permeability. In the present study, spraying plants with ZnO-NPs resulted in a significant reduction in MDA levels as well as EL, thus maintaining the structural integrity of membranes. Histochemical evaluation of leaf and root lipid peroxidation confirmed these results, as a strong pink color was observed in untreated samples, whereas much lighter coloring in treated samples indicated lower MDA levels. It means that high antioxidant enzyme activity helped scavenge excessive ROS, preventing their damaging effects on membranes. Reductions in MDA content after treatment with ZnO-NPs have previously been reported in tomato, maize (*Zea mays*), and mung bean (*Vigna radiata*), and the application of NPs prevented oxidative damage via improved antioxidant metabolism and membrane stability [47,54,55]. In recent studies on *B. juncea*, similar effects were observed with biosynthesized ZnO-NPs, including enhanced antioxidant potential and lower oxidative damage markers [41].

The application of ZnO-NPs had positive effects on several important parameters of chlorophyll fluorescence, including Fv/Fm, ΦPSII, ETR, and qP, whereas NPQ was reduced. This indicates improved use of light energy and reduced heat dissipation [46,56]. These results are consistent with earlier studies in which low concentrations of ZnO-NPs, such as 15–50 mg/L, sprayed on foliage increased chlorophyll content, ETR, ΦPSII, and qP in tomato and tea (*Camellia sinensis*) plants by enhancing light harvesting and PSII efficiency. The elevated Fv/Fm (optimal ~0.8) reflects intact PSII reaction centers and improved photosynthetic performance, as Zn supplementation aids enzyme function in electron transport [46]. Reduced NPQ suggests minimized photoprotective heat loss, allowing more energy for photochemistry, consistent with ZnO-NPs promoting Rubisco activity and pigment synthesis in stressed crops [46,56,57,58]. Conversely, doses of 75–100 mg/L caused declines in Fv/Fm, ΦPSII, ETR, and qP, alongside an elevation in NPQ, signaling PSII damage, closed reaction centers, and excess energy dissipation as heat. This dose-dependent toxicity aligns with findings in which higher doses of ZnO-NPs (e.g., >50 mg/L) induce ROS buildup, OEC impairment, and photoinhibition in *Vicia faba* and tomato, overwhelming protective mechanisms. Thus, application of ZnO-NPs at 50 mg/L resulted in the strongest enhancement of photosynthetic performance. However, increasing the concentration beyond this level progressively reduced the magnitude of the response, with noticeable declines at 75 and 100 mg/L. Such inhibition at higher exposure levels could be related to excessive Zn accumulation and disturbance of cellular Zn balance, although other concentration-dependent effects of ZnO-NPs may also contribute. Since the experiment did not include corresponding bulk ZnO or soluble Zn controls, the present data cannot establish whether the decline reflects conventional Zn toxicity or a response specifically associated with the nanoparticulate form of ZnO [58,59,60].

The increase in carbohydrates and reducing sugars at an optimal level of ZnO-NPs is consistent with previous reports, which have reported increases of 20–40% [53,61]. The observed carbohydrate enhancement could be attributed to the increased photosynthesis rate and related processes, including increased P_N_, ΦPSII, and ETRs. Increased sugar concentration increases carbon assimilation/translocation [59], but higher doses result in a decline in carbohydrate metabolism due to induced oxidative stress.

The increase in proline content following ZnO-NP application may reflect an adjustment of osmotic and redox metabolism in *B. juncea*. Proline is involved in several physiological processes, including osmotic regulation, stabilization of membranes and proteins, and modulation of reactive oxygen species homeostasis under stressful conditions [62,63]. In the present study, the highest proline content was observed at 50 mg/L ZnO-NPs, coinciding with lower ROS accumulation, reduced lipid peroxidation, and improved membrane stability. This association suggests that proline accumulation may have contributed to the overall redox adjustment observed at this concentration. However, because proline accumulation can occur as part of both adaptive and stress-related responses, it should not be considered independently as direct evidence of enhanced stress tolerance. Similar increases in proline metabolism following ZnO-NP application have been reported in other crop species [47,54,62,64]. In addition, the increase in reduced glutathione (GSH) observed in ZnO-NP-treated plants indicates an enhanced non-enzymatic antioxidant response. GSH is a major cellular antioxidant involved in ROS detoxification, maintenance of redox homeostasis, and protection of cellular macromolecules against oxidative damage [63]. The higher GSH content, particularly at 50 mg/L ZnO-NPs, was consistent with the lower ROS accumulation and reduced membrane damage observed in the present study. Previous studies have also reported stimulation of glutathione-dependent antioxidant metabolism following ZnO-NP treatment [47,65].

The high amounts of proteins may be explained by improved nitrogen assimilation and increased capacity of photosynthesis due to higher NR enzyme activity, along with other factors associated with photosynthesis and respiration in plants treated with ZnO-NPs. Nitrogen is an essential element in amino acids and proteins, and the function of NR is to oxidize nitrate (NO^3−^) into nitrite (NO^2−^). It is the first step of nitrogen assimilation, thus leading to the enhancement of NR activity in ZnO-NPs-treated plants, increasing nitrogen assimilation and utilization of organic nitrogen compounds that serve as precursors for proteins. Zinc ions play a critical role in energy transfers in plants; firstly, NR and many other enzymes participating in nitrogen metabolism and synthesis need Zn for protein synthesis. Zinc ions in ZnO-NPs enhance the bioavailability of Zn in plants; thus, NR, NiR, GS and GOGAT enzymes, which require Znss, are activated [16].

The increased photosynthetic performance (P_N_, C_i_, g_s_, E, Rubisco enzyme activity) and respiratory rate (SDH enzyme activity) would supply the energy (ATP) and carbon skeleton required for protein biosynthesis. Photosynthesis increases carbon fixation, which is used to produce organic building blocks (3-phosphoglycerate and oxaloacetate), which, when combined with nitrogen via transamination reactions, lead to the formation of amino acids. Studies by Chen et al. [46] indicate that the use of ZnO-NPs in tea plants improves photosynthesis and shoot growth, leading to increased biomass accumulation (and hence protein levels) due to increased chlorophyll content and efficiency. The combination of increased nitrogen metabolism (through NR enzyme activity) and increased photosynthesis/respiration (through Rubisco and SDH enzyme activities) provides a conducive biochemical milieu for protein synthesis, resulting in increased protein contents with the optimum use of ZnO-NPs at 50 mg/L.

Foliar application of ZnO-NPs altered the elemental composition of *B. juncea* leaves, with the highest relative values of Mg, S, P, K, Ca, and Zn generally observed in plants treated with 50 mg/L ZnO-NPs. These changes in leaf elemental composition were associated with improved physiological performance. Because elemental status was assessed using SEM–EDX, the present data should be interpreted as relative changes in leaf elemental composition rather than as direct measurements of whole-plant nutrient uptake or translocation. Additionally, the SEM-EDX analysis detected Zn in the leaf samples following ZnO-NP treatment, indicating the presence or association of Zn within the analyzed tissue. However, SEM-EDX alone cannot conclusively establish the uptake, internalization or translocation of intact ZnO-NPs, nor can it distinguish between nanoparticulate Zn and Zn present in the leaf tissues. Therefore, the observed Zn signal/effect is interpreted cautiously as evidence of Zn detection in the analyzed leaf samples rather than definitive confirmation of nanoparticle uptake or intracellular transport. Minerals play an important role in regulating photosynthesis, enzyme reactions, osmosis, and other physiological activities; thus, their higher concentrations in plants are associated with improved nutrition. Among the analyzed macronutrient elements, potassium showed the largest relative increase in plants treated with 50 mg/L ZnO-NPs compared with the control. Potassium plays an important role in osmotic regulation, stomatal function, maintenance of turgor, membrane potential, and enzyme activity [66,67]. The higher relative K abundance may therefore be associated with the improved stomatal and photosynthetic performance observed in ZnO-NP-treated plants. In addition, the role of potassium in enhancing phloem loading, ATP synthesis, and stress signaling pathways can be highlighted [68,69].

Moreover, there was a marked rise in the levels of magnesium due to the effect of ZnO-NPs. Magnesium is the metal at the center of the chlorophyll molecule that regulates light capture and carbon dioxide fixation during photosynthesis. Elevated magnesium levels might have facilitated the production of chlorophyll molecules and stimulated the observed increase in Rubisco activity in ZnO-NP-treated plants. Additionally, magnesium is a cofactor for various ATP-dependent enzymes involved in carbohydrate and protein synthesis [66,70]. The relative abundance of phosphorus was higher in ZnO-NP-treated leaves. Phosphorus is essential for energy transfer, the synthesis of nucleic acids and membrane phospholipids, and cellular signaling processes. This enhances cell division, metabolic activities, and biomass accumulation in plants. Higher phosphorus levels also enhance stress tolerance and root development by improving energy metabolism [67]. A higher relative Ca abundance was also observed, particularly at 50 mg/L ZnO-NPs. Calcium plays a role in stabilizing plasma membranes and cell walls, and serves as a secondary messenger in stress responses. Thus, the higher calcium accumulation could be attributed to reduced electrolyte leakage and improved membrane stability resulting from ZnO-NP exposure. Calcium-mediated pathways are known to regulate antioxidant defense mechanisms and development in plants [71].

Furthermore, the relative abundance of sulfur was also higher in ZnO-NP-treated leaves. Sulfur forms an important component of various sulfur-containing amino acids, glutathione, coenzymes, and defense enzymes. This sulfur accumulation could lead to glutathione production and improved antioxidant metabolism, thereby promoting ROS detoxification. Furthermore, sulfur deficiency in plants results in decreased enzyme activity and protein biosynthesis [72]. A higher relative Zn abundance was observed in leaves following foliar application of ZnO-NPs. However, because the elemental analysis was based on SEM–EDX, these data do not provide direct evidence of ZnO-NP uptake, Zn translocation, or whole-plant Zn accumulation. Zinc is a vital micronutrient required for hormone biosynthesis, membrane integrity, chlorophyll synthesis, and the function of many metalloenzymes. It is possible that increased Zn content could activate several physiological and biochemical processes, such as photosynthesis, antioxidants, and nitrogen metabolism [73].

Based on the observed yield parameters following treatment with 50 mg/L ZnO, it can be predicted that the optimum concentration of ZnO-NPs positively impacts the reproductive growth and productivity of *B. juncea*. The increase in pod number, seed number, seed weight, and overall production may be associated with improved photosynthetic performance, altered leaf elemental composition, enhanced enzyme activities, and greater biomass production in *B. juncea* plants. Zinc is important for pollen germination, protein synthesis, hormonal regulation, and reproductive physiology. It enhances plant yield and seed quality [28,45].

An important limitation of the present study is that DLS and zeta-potential measurements were not performed on the actual Tween-20-containing ZnO-NP spray suspension used for foliar application. Therefore, the hydrodynamic size, surface charge, and aggregation state of the nanoparticles under the actual application conditions could not be determined. Additionally, in the present study, the absence of bulk ZnO and soluble Zn controls, such as ZnSO_4_, which restricts our ability to distinguish responses attributable to Zn nutrition from those specifically associated with the nanoscale properties of ZnO-NPs. Although the tested concentrations produced clear dose-dependent physiological responses, the inhibitory effects observed at 75–100 mg/L cannot be conclusively assigned to nanoparticle-specific phytotoxicity and may instead reflect excessive Zn availability or other concentration-related effects. Furthermore, SEM-EDX analysis confirmed the detection of Zn in the examined leaf tissues following ZnO-NP application; however, this technique alone does not provide definitive evidence for the uptake, internalization or translocation of intact ZnO-NPs and cannot distinguish nanoparticulate Zn from Zn nutrition or other Zn-associated forms. Therefore, the SEM-EDX findings are interpreted conservatively as evidence of Zn detection or association within the analyzed leaf samples rather than direct confirmation of nanoparticle transport within plant tissues. Future studies incorporating bulk and soluble Zn controls, together with quantitative measurements of Zn accumulation, translocation and chemical speciation using appropriate analytical techniques such as ICP-MS/AAS, will be necessary to resolve these limitations and clarify the basis of the observed concentration-dependent responses.

## 4. Materials and Methods

### 4.1. Plant Sample and Nanoparticle Preparation

Seeds of *B. juncea* L. var. *Varuna* were obtained from a certified supplier in Aligarh, India. Uniform seeds were selected, surface-sterilized with 0.01% HgCl_2_, and subsequently rinsed several times with double-distilled water (DDW). ZnO-NPs (Product No. 721077-100G; CAS No. 1314-13-2) with a pH of 7.5 ± 1.5 were purchased from Sigma-Aldrich Chemicals Pvt. Ltd., Bengalore, India. A 100 mg/L ZnO-NP suspension was prepared in DDW and sonicated for 10 min. Aliquots of this suspension were subsequently diluted with DDW to obtain final concentrations of 25, 50, and 75 mg/L, while the undiluted 100 mg/L suspension was used as the highest-concentration treatment.

### 4.2. Nanoparticle Characterizations

The ZnO-NPs were characterized using X-ray diffraction [XRD; Rigaku SmartLab X-ray diffractometer (Rigaku Corporation, Akishima, Tokyo, Japan)], Fourier transform infrared spectroscopy (FTIR; PerkinElmer, Inc., Waltham, MA, USA), and scanning electron microscopy (SEM; JSM-6510 LV JEOL, Tokyo, Japan)) following the methodology described in our previous study [74]. The average crystallite size of the ZnO-NPs was determined by using the following Debye–Scherrer formula:

D = kλ/βCosθ

where D is the mean crystallite size, θ is the diffraction angle, X-ray wavelength is represented by λ, Scherrer’s constant is denoted by k, which is equivalent to 0.9 for spherical particles, and the k value relies upon the particle shape and diffraction indices (hkl). β is the full width at half maxima (FWHM) of the peak.

### 4.3. Experimental Setup

The experiment was conducted using a completely randomized design involving 20 earthen pots (10 inches), containing soil of sandy-loam texture enriched with farmyard manure in the proportion of 6:1. A common basal fertilizer dosage of inorganic nutrients like urea, single superphosphate, and muriate of potash was added in the amounts of 40 mg, 138 mg, and 26 mg per kg of soil, respectively.

Each treatment comprised four biological replicate pots (*n* = 4), with each pot considered one experimental unit throughout the experiment. Three plants were maintained in each pot after thinning, resulting in a total of 20 experimental pots and 60 plants across the five treatments. The same set of pots was used for sampling at both 30 and 45 DAS; thus, no independent sets of pots were established for the two sampling periods.

To facilitate destructive sampling at both time points, at 30 DAS, one plant from each pot was harvested for destructive measurements, while the remaining two plants were maintained in the same pots. At 45 DAS, one of the two remaining plants from each pot was harvested for subsequent measurements. The final remaining plant in each pot was maintained until 120 DAS and used for yield assessment. Thus, the same four replicate pots per treatment were maintained throughout the experiment, while individual plants within each pot were sequentially sampled at 30 and 45 DAS, followed by yield assessment of the remaining plant at 120 DAS. Thus, root and shoot length and fresh and dry biomass were determined from the plants harvested at the respective sampling dates and were considered destructive measurements. Physiological and photosynthetic measurements that did not require plant harvesting were performed non-destructively at the respective sampling times.

Foliar application of ZnO-NPs was initiated at 25 days after sowing (DAS). Fresh suspensions containing 25, 50, 75, and 100 mg/L ZnO-NPs were prepared in DDW containing 0.05% (*v*/*v*) Tween-20 as a surfactant and ultrasonicated for 10 min immediately before application to promote dispersion. Control plants were sprayed with an equivalent volume of DDW containing 0.05% (*v*/*v*) Tween-20 but without ZnO-NPs.

Plants were sprayed once daily during the afternoon for five consecutive days, applying approximately 3 mL of suspension per plant to ensure uniform foliar coverage. Control plants received an equal volume of DDW. The foliar application of ZnO-NPs was conducted by treating each plant with about 3 mL of the corresponding suspension for 5 consecutive days. The amounts of ZnO-NPs used per plant, based on the concentration and volume of suspension applied, was calculated as follows (Table 2).

These values refer to the nominal quantity of ZnO-NPs present in the sprayed solution and not to the amount deposited or absorbed in the plant.

All treatments were performed under uniform net-house conditions (20 ± 5 °C and 60 ± 5% relative humidity). At 30 and 45 DAS, various morphological, biochemical, photosynthetic, and antioxidant parameters were evaluated to assess the potential of ZnO-NPs to enhance the growth and performance of mustard plants. Yield traits were analyzed at 120 DAS. The treatment plan of the experiment is as follows:

- Group I: Control (sprayed with DDW containing 0.05% (*v*/*v*) Tween-20, without ZnO-NPs);
- Group II: Foliar application of 25 mg/L ZnO-NPs;
- Group III: Foliar application of 50 mg/L ZnO-NPs;
- Group IV: Foliar application of 75 mg/L ZnO-NPs;
- Group V: Foliar application of 100 mg/L ZnO-NPs.

Neither bulk ZnO nor soluble Zn salt treatment was used in the current experimental study. Hence, the present experiment aimed at studying the concentration effect of ZnO-NP formulation on *B. juncea* instead of determining the specific effects of nanoparticles of ZnO on the test plant. Treatment involving both bulk ZnO and soluble Zn compound at the same concentration of Zn was not considered within the current experiment.

### 4.4. Growth Analysis

The plants were taken out of their pots and were washed with water to remove dirt and soil. The lengths of the shoots and roots were recorded. Fresh weight of shoots and roots was recorded using an electronic balance. The oven-drying method was used to determine the dry weights of shoots and roots for each treatment by drying them in an oven at 80 °C for 24 h. The area of the leaf was estimated using a leaf area meter (ADC Bioscientific, Ltd., Hoddesdon, UK).

### 4.5. SPAD Index (Chlorophyll Content)

Chlorophyll meter (SPAD-502, Konica, Minolta Sensing, Inc., Tokyo, Japan) was utilized to determine the chlorophyll concentration in the leaf. The working principle of the chlorophyll meter relies on the absorbance of red and far-red light regions for determining the concentration of chlorophyll.

### 4.6. Morphology Study of Leaf Stomata

The protocol described by Siddiqui et al. [75] was used to visualize stomata on the leaf surface using SEM. Fresh leaf tissues of 50 mg/L ZnO-NPs and control of 45 DAS were collected and then fixed in 0.1 M sodium cacodylate buffer solution (pH 7.3), 2% paraformaldehyde, and 2.5% glutaraldehyde for 2 h. Subsequently, dehydration was performed by immersing the samples in ethanol at increasing concentrations (50%, 70%, 80%, 90%, and 100%). The fully dehydrated leaves were coated with gold–palladium using a sputter-coating apparatus (JOEL JFC-1600, Tokyo, Japan), and the stomatal apertures were then imaged by scanning electron microscopy.

### 4.7. Photosynthesis and Gas Exchange Characteristics

The gas exchange was performed using a portable photosynthesis system (LICOR 6400, Lincoln, NE, USA) with an infrared gas analyzer (IRGA). All the readings of P_N_, g_s_, E, and C_i_ were taken under strictly controlled conditions, which included a light intensity of 1200 µmol m^−2^ s^−1^, a CO_2_ concentration of 400 ppm, a leaf temperature of 25 °C, and a relative humidity of 60%. Gas-exchange measurements were conducted between 09:00 and 11:00 h using fully expanded mature leaves.

### 4.8. Chlorophyll Fluorescence Analysis

Measurements of chlorophyll fluorescence parameters were performed using a PAM fluorometer (PAM-200, Heinz Walz GmbH, Effeltrich, Germany). Fully grown leaves were dark-adapted for 20–30 min before analysis. Fluorescence at minimal (Fo) and maximal (Fm) levels was recorded under weak modulated light and a saturated light pulse, respectively, and the maximum quantum efficiency of PSII (Fv/Fm) was calculated. In the presence of actinic light, steady-state fluorescence (Fs) and maximal fluorescence in the light-adapted state (Fm′) were determined.

The effective quantum yield of PSII (ΦPSII) was calculated as follows:

ΦPSII = (Fm′ − Fs)/Fm′

where Fm′ = fluorescence at the maximum level in the light-adapted state and Fs = steady-state fluorescence. The electron transport rate (ETR) was calculated as follows:

Electron transport rate (ETR) = ΦPSII × PAR × 0.5 × 0.84

where ΦPSII = the effective quantum yield of PSII, PAR = photosynthetically active radiation, 0.5 = energy distribution between PSII and PSI and 0.84 = the leaf absorbance factor.

The photochemical quenching (qP) and non-photochemical quenching were calculated as follows:

photochemical quenching (qP) = (Fm′ − Fs)/(Fm′ − Fo′)

non-photochemical quenching (NPQ) = (Fm − Fm′)/Fm′

where Fm′ = fluorescence at the maximum level in the light-adapted state, Fs = steady-state fluorescence, Fo′ = fluorescence at the minimal level in the light-adapted state, and Fm = fluorescence at the maximum level in the dark-adapted state.

### 4.9. Total Carbohydrate Content and Sugar Analysis

The total amount of soluble and reducing sugars was estimated by extracting leaf powder with 80% ethanol solution. This was done for 50 mg of leaf material. The extracted material was then centrifuged at 10,000 rpm. Soluble sugars were estimated according to the procedure of Dubois et al. [76]. The reading was taken at 485 nm. Reducing sugars were estimated according to the procedure of Sumner and Howell [77]. The reading was taken at 560 nm.

### 4.10. Enzymatic Analysis

Enzyme assays were performed on freshly harvested leaves using conventional methods. Nitrate reductase (NR) activity was determined according to the method described by Jaworski [78]. Leaf material was incubated in a reaction mixture containing KNO_3_ and phosphate buffer, and the resulting absorbance was measured at 540 nm. The carbonic anhydrase (CA) activity was estimated by the titration method, as described by Dwivedi and Randhawa [79], using NaHCO_3_ and indicators. SOD activity was determined according to the method of Kono [80], based on nitroblue tetrazolium reduction, with absorbance measured at 560 nm. CAT activity was measured by decomposition of H_2_O_2_ and absorbance measured at 240 nm [81], whereas POX activity was estimated by the method of Chance and Maehly [82] with measurement of absorbance at 420 nm. Rubisco activity was determined using a coupled enzymatic assay based on RuBP-dependent NADH oxidation, following the method of Servaites and Torisky [83]. Succinate dehydrogenase (SDH) activity was determined according to the method of Kun and Abood [84]. Enzyme activities were expressed on a fresh-mass basis.

### 4.11. Proline Content Estimation

Proline concentration was estimated according to the method described by Bates et al. [85], with some modifications. Homogenate (0.25 g) prepared in 3% sulfosalicylic acid was centrifuged at 10,000× *g* for 10 min. To the 2 mL of supernatant were added 1 mL of acid ninhydrin reagent, 2.5 mL of sulfosalicylic acid, and 1 mL of glacial acetic acid. The reaction mixture was incubated at 100 °C for 1 h and then rapidly cooled in an ice bath to stop the reaction. Toluene was added, and the color complex formed was extracted. Readings were taken at a wavelength of 520 nm.

### 4.12. Glutathione Content Analysis

The concentration of reduced glutathione (GSH) was estimated according to the procedure outlined by Tietze [86]. Leaf tissues were first homogenized with 5% sulfosalicylic acid, and the mixture was centrifuged at 10,000× *g* for 10 min at 4 °C. The reaction mixture included phosphate-buffered solution with pH adjusted to 7.5, DTNB, NADPH, and glutathione reductase. An increase in absorbance due to the formation of yellow-colored 5-thio-2-nitrobenzoic acid (TNB) was measured spectrophotometrically at 412 nm. GSH concentration was obtained from a standard curve, and results were expressed as nmol g^−1^ FM.

### 4.13. Lipid Peroxidation Assessment

Lipid peroxidation was estimated using the method of Heath and Packer [87]. Fresh leaf tissue was homogenized in 0.1% TCA, and the homogenate was centrifuged at 10,000× *g* for 15 min. The resulting supernatant was mixed with 20% TCA containing 0.5% thiobarbituric acid. It was then incubated at 95 °C for 30 min and cooled to 4 °C. After incubation, the samples were rapidly cooled and centrifuged at 10,000× *g* for 15 min. The absorbance of the supernatant was recorded at 532 nm.

### 4.14. Total Protein Content

Protein content was determined using the Bradford [88] method. The absorbance was measured at 595 nm.

### 4.15. Confocal Laser Scanning Microscopy for Cell Viability Analysis in Roots

Cell viability assay was performed according to Rattan et al. [89]. Roots of the control and ZnO_50 treatments were immersed in a solution containing 25 µM propidium iodide for 10 min. The samples were examined using a Zeiss LSM 780 confocal laser scanning microscope at 20× magnification, using an excitation range of 535–543 nm and an emission range of 617–620 nm for PI fluorescence detection.

### 4.16. Histochemical Detection and Hydrogen Peroxide Estimation

H_2_O_2_ was localized using the protocol of Kaur et al. [90]. Leaves of *B. juncea* (treated with 50 mg/L ZnO-NPs, 45 DAS), and the control were immersed in 3,3-diaminobenzidine solution (DAB, pH = 3.8) for eight hours, followed by soaking and boiling in pure ethanol till complete chlorophyll removal. Leaves were cooled to room temperature and dipped in 20% glycerol, resulting in brown spots indicating hydrogen peroxide production. A picture was taken through a stereomicroscope. Hydrogen peroxide content was estimated following the protocol of Siddiqui et al. [75].

### 4.17. Histochemical Detection and Superoxide Anion Estimation

Superoxide anions (O_2_^•−^) were localized using a slightly modified protocol of Kaur et al. [90]. Leaves of *B. juncea* were immersed for 8 h at room temperature with adequate light in an NBT solution (6 mM in sodium citrate buffer) and then soaked and boiled in pure ethanol until complete chlorophyll removal. The leaves were cooled down to room temperature and dipped in 20% glycerol. A picture was taken through a stereomicroscope. Superoxide anion (O_2_^•−^) content was estimated following the method described by Siddiqui et al. [75].

### 4.18. Electrolyte Leakage

For each sample, electrolyte leakage (EL) was measured using a conductivity meter (CM-115, Kyoto Electronics, Kyoto, Japan) both before and after autoclaving at 121 °C for 20 min [91].

### 4.19. Elemental Composition Analysis

The elemental composition of leaf samples, including carbon (C), magnesium (Mg), phosphorus (P), sulfur (S), potassium (K), calcium (Ca), and zinc (Zn), was analyzed at 45 DAS using scanning electron microscopy coupled with energy-dispersive X-ray spectroscopy (SEM–EDX), following the procedure described by Sharma et al. [92].

### 4.20. Yield Attributes

The parameters for yield assessment were measured at harvest (120 DAS) by counting the number of pods per plant, the number of seeds per pod, the weight of 100 seeds, and the yield per plant, using the method described by Gomez and Gomez [93]. The number of pods was manually counted, and the number of seeds per pod was assessed by averaging the number of seeds from randomly selected pods. The weight of 100 seeds was measured using an analytical balance, and the yield per plant was determined as the total seed weight per plant.

### 4.21. Statistical Analysis

Statistical analyses were performed using IBM SPSS Statistics version 31.0.2.0 for Windows (IBM Corporation, Armonk, NY, USA). Each treatment comprised four biological replicate pots (*n* = 4), with each pot considered one experimental unit throughout the experiment, including both the 30 and 45 DAS sampling periods. Data are presented as mean ± standard error (SE). Differences among treatments were evaluated using one-way analysis of variance (ANOVA), followed by Duncan’s multiple-range test for mean separation. Differences were considered statistically significant at *p* ≤ 0.05. For parameters measured at both 30 and 45 DAS, treatment effects were analyzed separately within each sampling date using one-way ANOVA. As the same experimental pots were used at both sampling dates, treatment effects were analyzed separately at 30 and 45 DAS using one-way ANOVA. These analyses were restricted to treatment comparisons within each sampling date; no statistical inference was made regarding treatment × time interactions or differences between sampling dates. Graphs were prepared using SigmaPlot version 16.0.5.

## 5. Conclusions

This study demonstrates that foliar application of ZnO-NPs modulates growth, photosynthesis, antioxidant metabolism, redox homeostasis, and yield formation in *B. juncea* in a concentration-dependent manner. Among the tested doses, 50 mg/L was the most effective, improving biomass accumulation, SPAD index, gas exchange, PSII efficiency, carbohydrate metabolism, antioxidant defense and yield attributes. The reduction in H_2_O_2_, O_2_^•−^, MDA, and electrolyte leakage, together with increased SOD, CAT, POX, proline, and GSH, suggests improved redox balance and membrane stability. However, the beneficial response declined at 75–100 mg/L, emphasizing the importance of dose optimization when ZnO-NPs are considered as potential foliar inputs for *B. juncea*. These findings demonstrate the importance of dose optimization when ZnO-NPs are considered as potential foliar inputs for *B. juncea*. However, the present results were obtained under controlled net-house conditions and should not be directly extrapolated to field-scale application. The absence of bulk ZnO and soluble Zn controls limits the ability of the present study to distinguish nanoparticle-specific effects from responses associated with Zn nutrition or Zn availability. Future comparative studies incorporating these controls are therefore needed to clarify the specific contribution of the nanoparticulate form. Further studies should evaluate ZnO-NP performance under field conditions, compare nanoparticulate ZnO with conventional Zn sources, determine nanoparticle uptake and translocation, and assess potential long-term environmental consequences before large-scale agricultural use is considered.

## Figures and Tables

**Table 1:** Leaf elemental composition of *B. juncea* at 45 DAS as influenced by foliar application of different ZnO-NP concentrations and determined by SEM–EDX analysis. All data are represented as mean ± SE. Means with the same letters are not significantly different (*p* ≥ 0.05) according to Duncan’s multiple-range test.

Treatment	C [% wt] *	Mg [% wt]	S [% wt]	P [% wt]	K [% wt]	Ca [% wt]	Zn [% wt]
Control	33.208 ± 0.628 ^c^	0.340 ± 0.006 ^c^	0.450 ± 0.009 ^c^	0.730 ± 0.014 ^d^	2.211 ± 0.042 ^d^	1.180 ± 0.022 ^d^	0.0056 ± 0.00011 ^e^
ZnO_25	35.109 ± 0.664 ^abc^	0.361 ± 0.007 ^bc^	0.471 ± 0.009 ^bc^	0.790 ± 0.015 ^bc^	2.441 ± 0.046 ^bc^	1.290 ± 0.024 ^bc^	0.0068 ± 0.00013 ^c^
ZnO_50	36.709 ± 0.694 ^a^	0.385 ± 0.007 ^a^	0.507 ± 0.010 ^a^	0.850 ± 0.016 ^a^	2.711 ± 0.051 ^a^	1.400 ± 0.026 ^a^	0.0078 ± 0.00015 ^a^
ZnO_75	35.709 ± 0.675 ^ab^	0.372 ± 0.007 ^ab^	0.482 ± 0.009 ^ab^	0.820 ± 0.016 ^ab^	2.571 ± 0.049 ^ab^	1.350 ± 0.026 ^ab^	0.0072 ± 0.00014 ^b^
ZnO_100	34.509 ± 0.653 ^bc^	0.354 ± 0.007 ^bc^	0.460 ± 0.009 ^bc^	0.770 ± 0.015 ^cd^	2.361 ± 0.045 ^c^	1.250 ± 0.024 ^cd^	0.0063 ± 0.00012 ^d^

* % wt = EDX-derived elemental weight percentage.

**Table 2:** Applied ZnO-NP doses per plant by foliar spraying at different concentrations.

ZnO-NP Conc. (mg/L)	Volume per Application (mL plant^−1^)	No. of Applications	Nominal Amount of ZnO-NPs per Application (mg plant^−1^)	Cumulative Nominal Amount of ZnO-NPs (mg plant^−1^)
25	3	5	0.075	0.375
50	3	5	0.15	0.75
75	3	5	0.225	1.125
100	3	5	0.3	1.5

## Data Availability

The data presented in this study are available on request from the corresponding authors. The data are not publicly available due to privacy reason.

## Abbreviations

The following abbreviations are used in this manuscript:

Ag	Silver
ANOVA	analysis of variance
AsA	Ascorbate
AsA-GSH	ascorbate–glutathione antioxidant system
ATP	adenosine triphosphate
C	Carbon
CA	carbonic anhydrase
Ca	Calcium
CAT	Catalase
Ci	intercellular carbon dioxide concentration
CO_2_	carbon dioxide
DAB	3,3′-diaminobenzidine
DAS	days after sowing
DCFH-DA	2′,7′-dichlorodihydrofluorescein diacetate
DDW	double-distilled water
DNA	deoxyribonucleic acid
DTNB	5,5′-dithiobis(2-nitrobenzoic acid)
DW	dry weight
E	transpiration rate
EL	electrolyte leakage
ETR	electron transport rate
Fe_2_O_3_	iron(III) oxide
FM	fresh mass
Fm	maximum fluorescence in the dark-adapted state
Fm′	maximum fluorescence in the light-adapted state
Fo	minimum fluorescence in the dark-adapted state
Fo′	minimum fluorescence in the light-adapted state
Fs	steady-state fluorescence
FTIR	Fourier transform infrared spectroscopy
Fv	variable fluorescence
Fv/Fm	maximum quantum efficiency of photosystem II
FWHM	full width at half maximum
GOGAT	glutamate synthase (glutamine:2-oxoglutarate aminotransferase)
GS	glutamine synthetase
GSH	reduced glutathione
g_s_	stomatal conductance
H_2_O_2_	hydrogen peroxide
H^+^-ATPase	proton-translocating adenosine triphosphatase
HgCl_2_	mercury(II) chloride
IRGA	infrared gas analyzer
JCPDS	joint committee on powder diffraction standards
K	Potassium
KNO_3_	potassium nitrate
MDA	Malondialdehyde
Mg	Magnesium
NADH	reduced nicotinamide adenine dinucleotide
NADPH	reduced nicotinamide adenine dinucleotide phosphate
NaHCO_3_	sodium bicarbonate
NBT	nitro blue tetrazolium
NiR	nitrite reductase
NO_2_^−^	Nitrite
NO_3_^−^	Nitrate
NP/NPs	nanoparticle/nanoparticles
NPQ	non-photochemical quenching
NR	nitrate reductase
O_2_^•−^	superoxide anion
OEC	oxygen-evolving complex
P	Phosphorus
P5CS	Δ^1^-pyrroline-5-carboxylate synthetase
PAM	pulse-amplitude modulation
PAR	photosynthetically active radiation
PDF	powder diffraction file
PI	propidium iodide
PM	plasma membrane
PN	net photosynthetic rate
POX	Peroxidase
PSI	photosystem I
PSII	photosystem II
ΦPSII	effective quantum yield of photosystem II
qP	photochemical quenching
ROS	reactive oxygen species
Rubisco	ribulose-1,5-bisphosphate carboxylase/oxygenase
RuBP	ribulose-1,5-bisphosphate
S	Sulfur
SDH	succinate dehydrogenase
SE	standard error
SEM	scanning electron microscopy
SEM-EDX	scanning electron microscopy–energy-dispersive X-ray spectroscopy
SiO_2_	silicon dioxide
SOD	superoxide dismutase
SPAD	soil–plant analysis development
TCA	trichloroacetic acid
TiO_2_	titanium dioxide
TNB	5-thio-2-nitrobenzoic acid
XRD	X-ray diffraction
Zn	Zinc
ZnO	zinc oxide
ZnO-NP/ZnO-NPs	zinc oxide nanoparticle/zinc oxide nanoparticles
ZnO_25/ZnO_50/ZnO_75/ZnO_100	treatment codes denoting 25, 50, 75, and 100 mg/L ZnO-NPs, respectively
